# Early heart disease prediction using LV-PSO and Fuzzy Inference Xception Convolution Neural Network on phonocardiogram signals

**DOI:** 10.3389/fninf.2025.1655003

**Published:** 2025-10-01

**Authors:** D. Prabha Devi, C. Palanisamy

**Affiliations:** 1 Department of Computer Science and Engineering, Bannari Amman Institute of Technology, Sathyamangalam, Tamil Nadu, India; 2 Department of Information Technology, Bannari Amman Institute of Technology, Sathyamangalam, Tamil Nadu, India

**Keywords:** heart disease, phonocardiogram signals, feature dimensions, swarm optimization, fuzzy inference, Xception CNN, diastolic and systolic differences, non-linear scaling

## Abstract

**Introduction:**

Heart disease is one of the leading causes of mortality worldwide, and early detection is crucial for effective treatment. Phonocardiogram (PCG) signals have shown potential in diagnosing cardiovascular conditions. However, accurate classification of PCG signals remains challenging due to high dimensional features, leading to misclassification and reduced performance in conventional systems.

**Methods:**

To address these challenges, we propose a Linear Vectored Particle Swarm Optimization (LV-PSO) integrated with a Fuzzy Inference Xception Convolutional Neural Network (XCNN) for early heart risk prediction. PC G signals are analyzed to extract variations such as delta, theta, diastolic, and systolic differences. A Support Scalar Cardiac Impact Rate (S2CIR) is employed to capture disease specific scalar variations and behavioral impacts. LV-PSO is used to reduce feature dimensionality, and the optimized features are subsequently trained using the Fuzzy Inference XCNN model to classify disease types.

**Results:**

Experimental evaluation demonstrates that the proposed system achieves superior predictive performance compared to existing models. The method attained a precision of 95.6%, recall of 93.1%, and an overall prediction accuracy of 95.8% across multiple disease categories.

**Discussion:**

The integration of LV-PSO with Fuzzy Inference XCNN enhances feature selection aPSO with Fuzzy Inference XCNN enhances feature selection and nd classification accuracy, significantly improving the diagnostic capabilities of PCG-classification accuracy, significantly improving the diagnostic capabilities of PCG-based systems. These results highlight the potential of the proposed framework as a based systems. These results highlight the potential of the proposed framework as a reliable tool for early heart disease prediction and clinical decision support.reliable tool for early heart disease prediction and clinical decision support.

## 1 Introduction

One of the leading causes of death globally is heart disease, impacting human life due to clinical identification errors resulting in increased fatalities. Early prediction and data analysis are essential in reducing the risk of patient outcomes and efficiently analyzing data (Bourouhou et al., 2019; Vahanian et al., 2021). Most existing techniques must concentrate on the disease properties and feature dimension in the intake data analysis structure (Er, 2021). So, increasing variations in feature analysis takes more dimension to produce poor accuracy in the sense of low precision, recall rate, and F1 measure on various parameters.

By considering the problematic issues, the optimization must improve the feature selection and classification for extraordinary outcomes (Schmidt et al., 2010; Deng et al., 2020). Cardiovascular disease is the leading cause of death worldwide, affecting millions of people annually with a variety of heart conditions. Patients must receive timely and efficient treatment for heart disease if early detection and correct diagnosis are to be achieved. Furthermore, Machine Learning (ML) algorithms provide significant assurance in clinical diagnosis, specifically in heart sound classification for diagnosing cardiac diseases (Shuvo et al., 2021).

Furthermore, many abnormalities, such as heart murmurs and artifacts associated with cardiovascular disease, can affect the heart rate, which is the most common cause of death. Thus, they offer a new method for early detection of heart disease (Shuvo et al., 2023; Dhiyanesh et al., 2024). Afterward, feature vectors can be generated by extracting features from the inputs acquired directly from the heart sounds using a Deep Neural Network (DNN) algorithm. Moreover, the efficacy of the suggested approach can be evaluated in a real-world setting (Springer et al., 2015).

**Figure 1:**
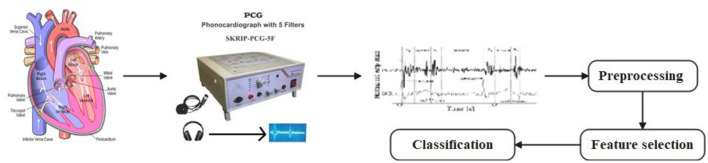
Working principle PCG signal observation and processing.

A promising method for classifying heart sounds involves analyzing recordings of sounds created by the heart during each cardiac cycle using PCG signals. Figure 1 describes the Working Principle of PCG Signal Observation and Processing. These signals contain valuable information about heart function and can be analyzed using DL techniques to identify patterns associated with different heart states (Waaler et al., 2023). In this paper, we present future work to improve the classification of heart diseases by utilizing a combination of Linear Vector Particle Swarm Optimization (LVPSO) and Xception Convolutional Neural Networks (XCNN). In the first step, the PCG signal can be pre-processed using the proposed methods to extract the relevant features for classification. This includes techniques such as signal elimination, segmentation, and feature extraction to improve data quality and reduce noise and artifacts that can interfere with classification. Once the data is pre-processed, it can be input into the LVPSO algorithm. The LVPSO algorithm is a variation of the traditional particle swarm optimization algorithm designed for linear vector optimization problems.

The LVPSO algorithm works by repeatedly updating the number of candidate solution particles based on the fitness values of the sound signal, which are determined by a linear vector objective function. This enables the algorithm to search the solution space efficiently and find the optimal parameters for the classification task. Using LVPSO, hyperparameters of Xception CNN, such as learning rate, block size, and number of layers, can be effectively modified to increase the accuracy of the classification model. Xception CNN, a DL framework, has demonstrated outstanding performance in classification tasks and has recently been utilized in clinical signal analysis with optimistic results. By combining Xception CNN with LVPSO, we aim to leverage the strengths of both algorithms to improve the accuracy and robustness of cardiovascular disease classification models. This collaborative approach, where the Xception CNN will be trained on the pre-processed PCG signals to understand the underlying patterns associated with different heart states and the LVPSO will optimize the CNN, invites all of us to be part of this exciting journey toward better understanding and classifying cardiovascular diseases hyperparameters.

The proposed acoustic classification of cardiac disease using LVPSO and exception CNN has the potential to significantly enhance the accuracy and efficiency of cardiac disease diagnosis. By leveraging the power of ML algorithms and DL frameworks, a robust and reliable classification model can be developed to assist healthcare professionals in the early detection and treatment of cardiac disorders. However, it is crucial to note that further research and experiments are essential to validate the efficacy of this approach. The initial results are promising, suggesting that this approach could have a significant impact on the field of heart disease.

The paper is structured into several sections to outline the cardiac sound classification research process. Section 1 provides an introduction to the research; Section 2 reviews the principles of existing methods along with their pros and cons; Section 3 explains the proposed method; Section 4 presents method comparisons; and Section 5 concludes with a discussion and final remarks, highlighting the performance of the proposed work and suggesting future developments.

## 2 Related work

A recent literature review (Krishnan et al., 2020) comprehensively summarizes current research on using PCG signals in predicting cardiac diseases through ML and DL methods. The potential of advanced technologies like ML and artificial intelligence to significantly enhance the precision and effectiveness of cardiac disease prediction is gaining momentum. Cardiovascular disease, a leading global cause of mortality, underscores the importance of timely identification for successful intervention and prevention. Recent analyses through various signal transformations have highlighted the importance of predicting heart disease by audio signals (Kiranyaz et al., 2020). Analyzing heart sounds with a phonocardiograph allows for recording heart sounds, which can be further explored with computational algorithms.

Early identification of abnormal heart sounds is a significant challenge in predicting heart disease. Conventional diagnostic methods for heart disease, like Electrocardiograms (ECG), are usually invasive and require specialized approaches. However, the use of non-invasive tools like stethoscopes to gather sound signals offers a convenient and promising approach to predicting heart disease.

Recent technological advances have enabled the characterization of DL heart disease based on PCG signals (Dhiyanesh et al., 2025). Modern DL methods, such as CNNs and Recurrent Neural Networks (RNNs), have successfully identified inconsistencies in PCG data. The potential of these advanced methods to revolutionize early detection and diagnosis of heart disease, reduce the burden on healthcare facilities, and improve patient outcomes is significant. However, a substantial problem with PCG signal processing is the different limitations on feature selection. The dimensionality of breeding features often leads to incorrect feature selection and poor accuracy.

The novel proposed that the prediction accuracy of heart failure can be improved by combining neural networks and Particle Swarm Optimization (PSO) techniques. However, cardiovascular disease continues to be a significant issue globally, with its mortality rate on the rise (Mahalakshmi and Rout, 2023).

Moreover, the enhanced PSO algorithm identifies the optimal features and feature subsets. The optimal feature subset is carefully selected and fed into an ensemble classifier to determine the likelihood of heart disease accurately (Yuliandari et al., 2024). A new approach to a Neural Fuzzy Inference System (NFIS) for representing training data can be created using n-dimensional functions. NFIS optimizes learning algorithms by calibrating them with an error calculation module (Jha et al., 2022). The new approach aimed to detect cardiac disorders using health metrics gathered from wearable sensors integrated with a Fuzzy Logic Inference System (FLIS) (Kadu et al., 2022).

Furthermore, CNNs are extensively employed to predict heart disease in various domains, including computer vision and image identification. CNNs can accurately assist in analyzing and predicting heart disease. Furthermore, CNNs can automatically learn hierarchical representations of data (Alzubaidi et al., 2021).

PSO-based methods can be applied to optimize the parameters of stacked sparse autoencoders. Furthermore, PSO optimization permits enhancing the performance of feature learning and classification (Mienye and Sun, 2021).

Table 1 presents DL techniques, datasets, and methods derived from previous approaches for heart disease detection, outlining the constraints and accuracy of performance evaluation achieved in predicting heart diseases.

**Table 1:** Heart disease detection based on deep learning (DL) technique.

**References**	**Classification method**	**Dataset**	**Limitation**	**Performance evaluation**	**Accuracy**
Naveenkumar et al. (2022)	CNN-based Xception Network (CNN-XN)	Heart sound	The number of deaths caused by CVD is on the rise across the globe.	Accuracy, precision	94.52%
Nwonye et al. (2021)	CNN	Coronary heart disease	Inactivity and unhealthy fitness can also increase the risk of CVD	Sensitivity, accuracy	85.79%
Fu et al. (2020)	Multi-Scale CNN with Attention Mechanisms (MSCNN-AM)	Benchmark datasets	The blood vessels exhibit changes in their shape and show reduced variability.	Specificity, F1-score	0.83%
Gárate-Escamila et al. (2020)	Chi-square- principal component analysis	UCI ML repository	However, the classification of CVD can often be unbalanced.	Matthews correlation coefficient	85.67%
(Yang and Guan 2022)	Synthetic Minority Overestimation Technique (SMOTE)	Heart disease	CVD reduces the accuracy and effectiveness of clinical diagnostic data.	False positive rate, true positive rate	92.44%

The proposed method utilizes SMOTE to manage imbalanced data in datasets effectively. Besides, these permit accurate classification of a given dataset and ensure maximum accuracy in performance evaluation results (Waqar et al., 2021). Cardiac signals can be automatically detected by decomposing them into discrete model functions utilizing the Complete Ensemble Empirical Mode Decomposition (CEEMD) method (Manuel Centeno-Bautista et al., 2023). Moreover, the signal-to-noise ratio model parts can be approximated to extract time and frequency details of the decaying mode through the EEMD analysis method (Zhao et al., 2023). Hence, the Least Mean Square (LMS) algorithm offers an optimal adaptive filter system for accurately estimating noisy signals. Likewise, a noisy signal can be processed in series with multiple adaptive filter stages (Hannah Pauline and Dhanalakshmi, 2022).

Furthermore, DL techniques have analyzed the ability to predict heart disease from sound signals. For example, Raza et al. (2019) developed a CNN-based model that detects heart murmurs from acoustic signals with up to 90% accuracy. Similarly Jones et al. (2019), used RNN to predict the onset of atrial fibrillation with an accuracy of 85%. Furthermore, DL techniques such as CNN and RNN have indicated accurate results in analyzing sound signals to predict heart disease. While RNNs are more effective at collecting temporal correlations in data, CNNs are better at extracting spatial features from sound recordings. By combining the two techniques, researchers achieved greater accuracy in predicting various heart diseases. However, feature dimensionality creates worst-case scenarios during classification, as threshold changes in feature ranges can lead to lower precision and recall.

In addition to CNNs and RNNs, auto-encoders and Generative Adversarial Networks (GANs) are other DL techniques studied to predict cardiac disease in sound signals. For example Ramkumar et al. (2024), proposed a GAN-based model for generating artificial heart sounds to improve the training data and prediction accuracy. However, the parametric performance reduces the accuracy and results in a high error rate due to high time complexity and uncorrelated feature analysis.

The literature on predicting cardiac disease using DL techniques on sound signals still needs to be improved. Furthermore, these models focus more on large-scale analysis of populations in real-world clinical settings to ensure their effectiveness. In Amiriparian's et al. (2019) study, the authors proposed a DL model to classify heart sounds into different categories, including standard and abnormal. The model achieved high accuracy in differentiating various types of heart sounds and demonstrated the potential of DL in analyzing sound signals for heart disease prediction. Another study by Li H. et al. (2020) focused on using DL for early detection of heart murmurs. The authors have developed a DNN that can accurately classify heart murmurs based on acoustic signals, showing promising results for early diagnosis of heart disease.

In a review by Wang J. et al. (2018), the authors discussed the various DL techniques used in heart disease prediction, including CNNs and RNNs. The review highlighted the crucial role of sound signals in improving the accuracy of prediction models, ensuring the audience is well-informed about the key factors in heart disease detection.

Among the seminal works in the field (Yang et al., 2021) proposed a DL model for cardiac disease prediction using acoustic signals conducted. The results demonstrate a capable accuracy in diagnosing heart disease and highlight the potential of the DL technique. Based on sound signals, a DL model for the prediction of cardiac disease was established by another critical analysis (Li Y. et al., 2020). The author combined a CNN with a Long Short-Term Memory (LSTM) algorithm to accurately predict cardiac illness by analyzing auditory data. Furthermore, they demonstrate the effectiveness of combining different DL frameworks to improve forecasting performance. In addition to these studies, several research papers have investigated using DL techniques for heart disease prediction using audio signals. For example, Wang H. et al. (2018) proposed an RNN-based DL model for heart disease prediction, and Gomathi et al. (2024) used a hybrid DL model combining CNN and SVM for the same objective.

Furthermore, researchers have analyzed using transfer learning in heart disease prediction with sound signals. For example, Hettiarachchi et al. (2017) applied knowledge from pre-trained DL models to enhance heart disease prediction performance, showcasing the potential of transfer learning in this area. Bentley et al. (2011) assembled a PASCAL dataset of heart sounds from patients with and without heart disease and used a CNN for sound signal classification. The University of Michigan Health System presents the Murmur Database (MHSTP), comprising 23 heartbeat recordings computing 1496.8 s. In the CEEMD, murmurs in heart sound signals are detected. CEEMD is University of Michigan Health System (2015) more advanced than EMD as it solves the mode mixing issue present in EMD. Extraction of the murmur and heart sounds using composting methods such as EMD has been performed (Oliveira et al., 2021; Dhiyanesh et al., 2024). In general, Ali et al. (2023) using DL methods to examine sound signals for predicting cardiac diseases shows significant potential in enhancing early detection and treatment results, thereby improving patient outcomes. By employing artificial intelligence capabilities, researchers can create more precise and effective predictive models to support healthcare providers in delivering improved care to individuals with heart diseases.

Table 2 shows the proposed method derived from previous studies, describing its limitations and limitations. Furthermore, they can be tested in feature selection methods for predicting heart disease. The techniques listed in the table provide a systematic approach to selecting relevant features important for accurate heart disease prediction.

**Table 2:** The research gap in the feature selection method used for predicting heart disease.

**References**	**Research gap**	**Methodology**	**Feature used**	**Problems**
Dewangan et al. (2008)	Only covers time depends on the feature, not the actual limits of the feature	Discrete Wavelet Transform (DWT)	Time series DWT	ECG recordings are insufficient to reveal valve health information.
Schanze (2017)	ML and DL concepts for feature evaluation generate errors	Singular Value Decomposition (SVD)	SVD, mean filters	Transformation methods eliminate unwanted signal components
Othman and Khaleel (2017)	Time feature limits discover the coefficient dependencies of feature limits.	Fast Fourier Transform (FFT)	Shanon energy, DWT	Discovering the most efficient approach will require a significant amount of time.
Martinek et al. (2017)	Non-real feature limits cannot take the acoustic values	LMS	Adaptive mean filters and statistical features	A standard channel is necessary for fiber optic interferometry.
Rao (2019)	All feature limits and dimension forums	Finite Impulse Response (FIR)	Bimedical signal peak signal tupe features	However, measuring digital signals as discrete signal phases, such as time or amplitude, is necessary.
Sh-Hussain et al. (2016)	Statistcal part of frequencies are missing absolute values	Mel-Frequency Ceptrum Coefficients (MFCCs)	Alpha feature limits	CVD is one of the most severe illnesses that can lead to death.
VenkataHari Prasad and Rajesh Kumar (2015)	PCG signals are not supported	DWT	Time domain features	An ECG recording alone cannot provide information regarding the health of the valves.
Pan et al. (2015)	Real-time dataset series are not supported. Unconsistent margins are taken, and actual values are uncovered.	Backpropagation Algorithm (BPA)	Wavelet features	Low-level data on the determination
Lubaib and Ahammed Muneer (2016)	Least level margins of signals only support	K Nearest Neighbor (KNN)	Subset carinal features	Interpreting a PCG typically necessitates a proficient and seasoned practitioner.
Zubair (2021)	Utilizing the publicly available PhysioNet/Cinc Challenge 2016 database.	Multi-Layer Perceptron (MLP)	Mel frequency Cepstral Coefficients (MFCC)	Computational complexity rises when heart sounds are classified as normal or abnormal.

Some effective models can classify PCG signals using Attention-Based Bidirectional LSTM (A-BLSTM) techniques (Prabhakar and Won, 2023). Another study used (Sivakami and Prabhu, 2023) Cuckoo Search Bio-inspired Algorithm (CSBA) with DBN method for heart disease prediction. Similarly, the novel Muthulakshmi and Parveen (2023) developed a *Z*-score normalization, African Buffalo Optimization (ABO) methods for effective disease prediction. Study Taylan et al. (2023) concentrated on classification of cardiovascular disease with the help of support vector regression (SVR) and ANFIS algorithm. Similarly, the article Thakkar and Agrawal (2023) used a deep CNN and min-max normalization method. The novel Yusuf Ilu and Prasad (2023) introduced an autoregressive integrated moving average (ARIMA) and K-means Clustering methods for disease identification. The literature review indicates a rising interest in applying DL techniques for predicting cardiac disease based on audio signals. Various studies reviewed in this research have demonstrated the efficiency of DL models such as CNN, LSTM, and RNN in accurately predicting cardiac disease from audio signals. Besides, investigating transfer learning and hybrid models exhibits potential for further advancement in this field. In conclusion, exploring heart disease predictions through DL techniques using sound signals holds great promise in improving early detection. Through employing the capabilities of DL, researchers can create precise and effective predictive models that have the potential to save lives.

### 2.1 Problem identification factors and consideration

From the literature, we found the complex nature of heart disease prediction based on sound signals having difficulties.

- One of the critical issues in PCG signal processing is the potential for improper feature selection due to identical feature dimensions. This can significantly undermine the accuracy of the results, leading to poor outcomes. Feature dimensionality creates worst-case scenarios during classification because the range variation in feature ranges causes low precision and recall rates.
- The previous methods' simulation parameters degraded the performance accuracy, so it has a higher false rate due to non-relation feature analyses, more time complexity, and higher error rates.

### 2.2 Research gap

- A significant research gap exists in the understanding of complex features extracted from phonocardiogram (PCG) signals that can be used to predict cardiac disease.
- Including only time depends on the feature, not the actual limits of ML and DL concepts for feature estimation.
- The previous algorithms are insuufficiently focus herat disease early stage prediction and One of the reaserch gap in cardiovascular disease diagnosis is the quality of the analytical data. ECG and PCG signals are sensitive to noise and artifacts. The amount of data generated can be enormous, making it difficult to effectively manage this data for signal processing and interpretation, and researchers are continuously working to develop powerful techniques to reduce noise and improve signal quality.
- Missing data values will result in errors; Valueless data is fuzzy because it can be either true or false. Decision-making ability depends on the quality of data. Small improvements in data dimension can lead to large improvements in decision-making information.

## 3 Proposed methodology

Toward developing a Linear Vectored-Particle Swarm Optimization based on Fuzzy Inference Xception Convolution Neural Network for early heart risk prediction. The first step in this approach is to utilize the Pascal dataset, which contains valuable information in the form of PCG representation. PCG signal format is used to convert sound waves into data, allowing for the identification of critical features such as Delta, Theta, diastolic, and systolic differences present in the dataset. These factors significantly influence the risk of heart disease. The model accuracy is improved by applying preprocessing techniques such as SMOTE and EDAMF to cardiac clinical data. These techniques help normalize the data and address balances or inconsistencies present in the dataset, ultimately improving the overall model's overall performance.

**Figure 2:**
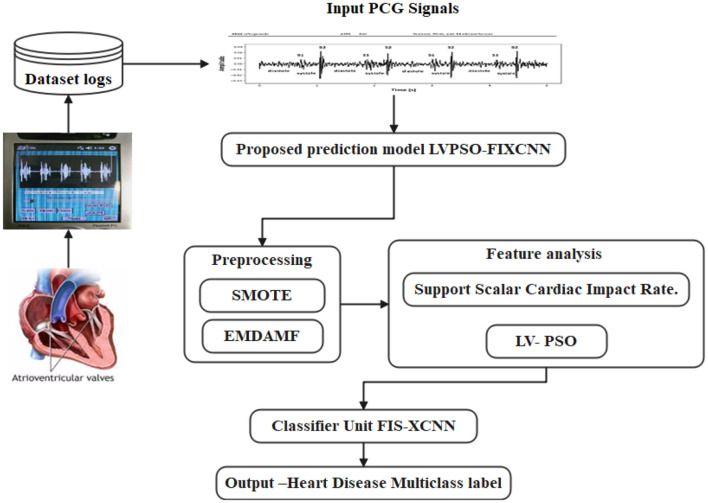
Proposed LVPSO-FIXCNN workflow architecture diagram.

To identify the scalar differences based on disease properties and assess the behavioral impact, a Support Scalar Cardiac Impact Rate (S2CIR) is utilized. This metric helps understand the disease's severity and impact on the individual, providing valuable insights for early detection and intervention. Figure 2 shows the Proposed LVPSO-FIXCNN Workflow Architecture Diagram. Notably, the Multivariate disease impact rate is used to determine the non-linearity scaling values, a crucial step in our research. These values are then processed using Linear Vectored–Particle Swarm Optimization (LV-PSO) for feature selection and dimensionality reduction, enhancing the model's performance and ensuring that only the most pertinent features are utilized for predictions.

Finally, the selected features are trained using a Fuzzy Inference Xception Convolution Neural Network (FIXCNN) to categorize the type of heart disease and provide accurate predictions. FIXCNN models utilize the capabilities of DL and fuzzy logic to examine intricate patterns in data and generate well-informed decisions, resulting in enhanced precision and dependability of predictions.

**Figure 3:**
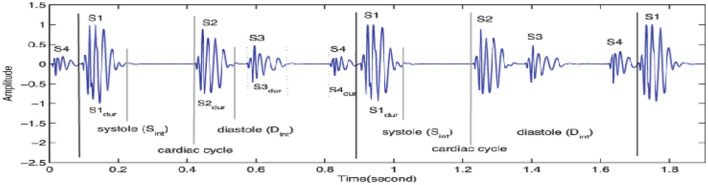
Heart sound PCG amplitude signal.

The heart PCG signals consist of several frequency components corresponding to different cardiac cycle physiological events, as indicated in Figure 3. The frequency range of cardiac sound waves is displayed in Table 3. The closure of the tricuspid and mitral valves produces the first heart sound (S1), which has low-frequency features. The closure of the aortic and pulmonary valves results in the second heart sound (S2), which has a higher frequency component.

**Table 3:** Levels of heart sound signal frequency limits.

**Frequency of sound signal levels**	**Frequency limits in Hz**		
Initial amplitude sector	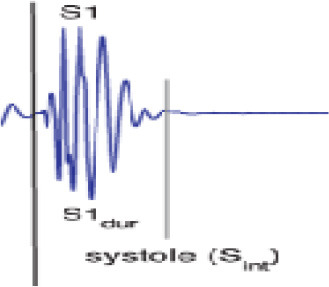	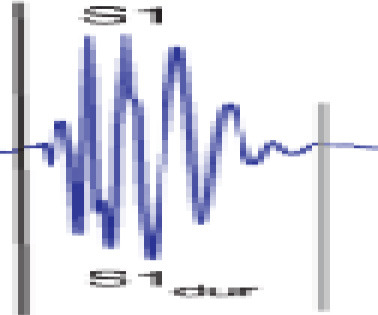 [S1]	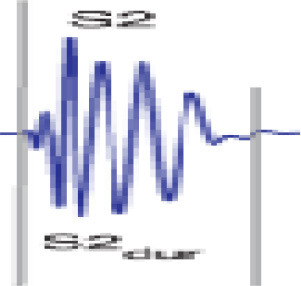 [S2]
Max-frequency energy strength	Systolic signal variation	≤ 30++, ≤ 100 Hz	100≥, ++ Hz
Post amplitude sector	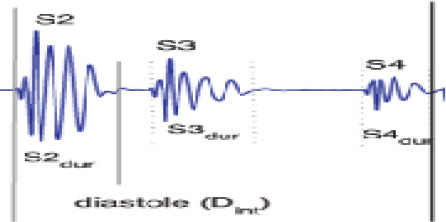	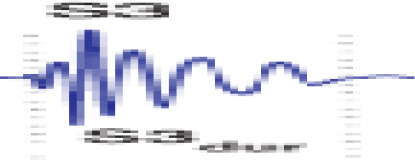 [S3]	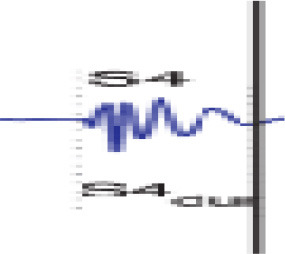 [S4]
Min frequency energy strength	Diastolic signal variation	20 ≤ , 25 Hz	≥30 Hz

Two additional heart sounds, S3 and S4, may indicate abnormal heart activity. The timing and intensity of cardiac PCG signals provide valuable information on the cardiac cycle. The heart sounds S1 to S2 are called the systolic interval, indicating the ventricular contraction and ejection length. The intensity of heart sounds may fluctuate due to factors like ventricular contraction and valve abnormalities. Changes in timing and intensity can indicate conditions such as heart failure or valvular stenosis.

### 3.1 Synthetic Minority Oversampling Technique (SMOTE)

In this section, a training dataset is developed using SMOTE to predict heart disease. Furthermore, leveraging the SMOTE technique can find extensive applications in the healthcare sector for managing class-imbalanced data. Then, by utilizing Euclidean distance to create synthetic generated random data of minority classes from nearby neighbors, the number of data instances can be enhanced. Moreover, new samples are generated by leveraging the top features from the original data. The SMOTE technique can produce optimal values of the application, thereby introducing additional noise. By oversampling minority classes, synthetic samples are created by adding line segments from the k nearest neighbors of the minority class to each sample. Neighbors can be selected randomly from the k nearest neighbors based on oversampling as required. A synthetic model is also created to predict the differences between the analyzed feature vector model and its nearest neighbors. Moreover, the feature vectors are evaluated with 0 and 1, multiplying their variances by random numbers.

Creating synthetic data from minority classes of random number data can be achieved by calculating population functions. Furthermore, nearest neighbors provide new index array features for different samples, as detailed in Algorithm 1. Let's assume *the i*_*d*_-number of the synthetic sample, Z-Minority instance, K-nearest neighbor, x- integral sample, *d*^*u*^−number of the attribute, W-sample, *q*−populate, W_*w*_−synthetic sample, D_*d*_−nearest neighbor, *d*_*x*_−new index, *x*_*u*_−attribute index, α−random number.

**Algorithm 1:**
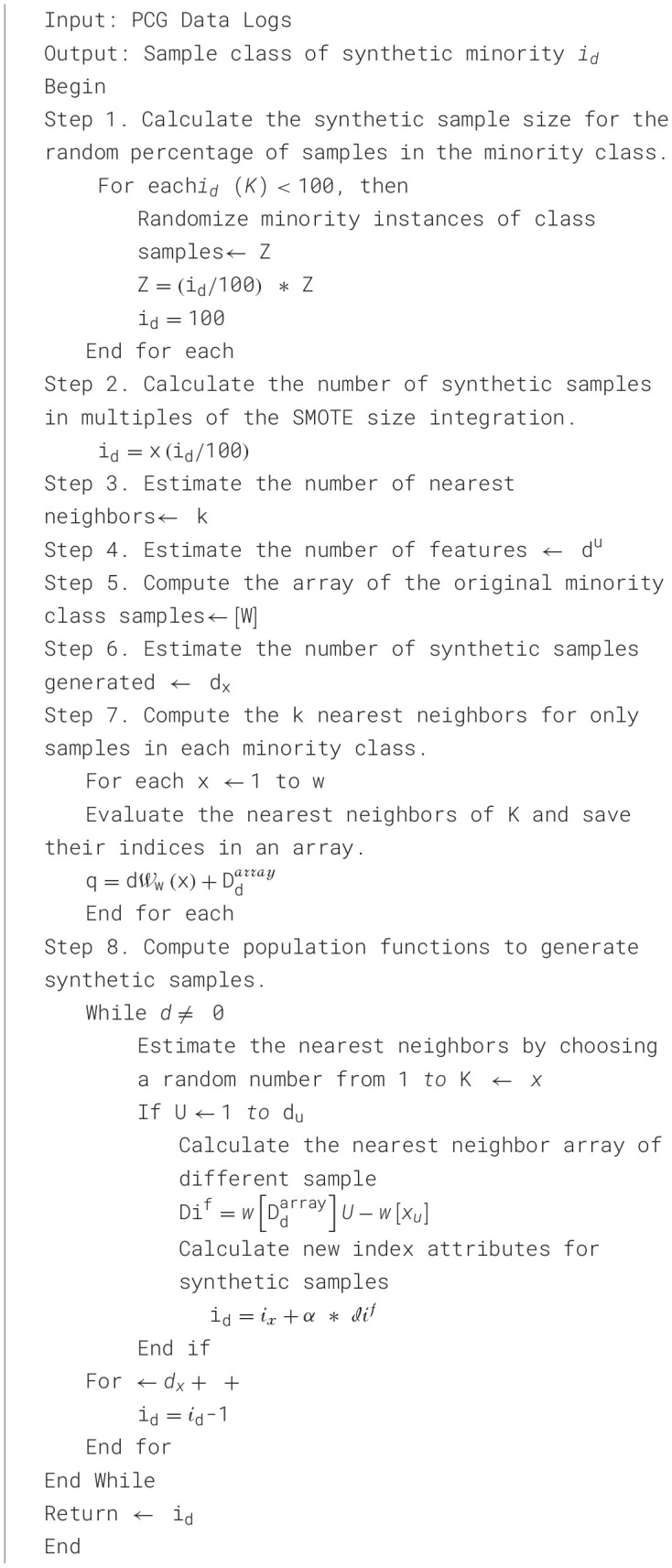
SMOTE.

Table 4 compares the performance of different variation methods like Support Vector Regression (SVR) and Autoregressive Integrated Moving Average (ARIMA). The proposed method attains 80.98%, 84.09%, and 90.21% for Pascal, Circor and Physico-cardnet, respectively.

**Table 4:** Comparison performance for variations methods.

**Methods/datasets**	**SVR**	**ARIMA**	**SMOTE**
Pascal	67.15	74.09	80.38
Circor	73.62	78.10	84.09
Physico- cardnet	79.35	80.54	90.21

### 3.2 Enhanced empirical mode decomposition adaptive filter

In this section, advanced empirical methods enable the analysis of time-domain or one-dimensional signals through an adaptive filter technique. The EEMDAF method is also known for decomposing a one-dimensional signal into different Eigenmode functions and frequency bands using frequency information. Also, the number and intensity of zero crossings in the 1D signal in the intrinsic mode functions must be different or equal. The estimated mean value will be zero when using symmetrical lower and upper envelopes. The process is repeated until all accurate Eigenmode functions are computed using the analytical EEMDAF method.

Moreover, filter integration approximates the input-output relationship of the EEMDAF method. By considering only current and past observations, the weighting of adaptive filters can produce statistically better estimates of the following observations.

Furthermore, the EEMDAF technique removes reference signal interference from the cross-correlation matrix and ensures vector independence. Noise estimation also includes estimating signals from the power supply and other known noise sources. Unlike frequency-selective filters, adaptive filters use an autocorrelation matrix instead of a crucial input to normalize the most and least significant engine values.

Lower and upper envelopes are estimated using cubic splines connecting the determined local maximum and minimum points, as described in Equation 1. Let's assume ${the\;k}_{1}^{z}-$new 1D signal, the *i*_*z*_−minimum and maximum point of the 1D signal, and the $c_{1}^{z}-$mean of both envelopes.


(1) $$\begin{array}{l} k_{1}^{z}={\text{i}}_{\text{d}}\left(i_{z}-c_{1}^{z}\right) \end{array}$$

Equation 2 approximates the new 1D signal's local maximum and minimum points. Compute the residual signal subtracted from the 1D signal and evaluate the Eigen mode function as shown in Equation 3. Where $e_{1}^{z}-$residual signal, IMF_1_−intrinsic mode functions, $m_{1}^{z}-$condition signal.


(2) $$\begin{array}{l} m_{1}^{z}=k_{h}^{z}=IMF_{1} \end{array}$$

(3) $$\begin{array}{l} w_{1}^{z}=i_{z}-{IMF}_{1} \end{array}$$

Calculate the final residual signal of the Eigen mode function derived from the initial 1D signal, as indicated in Equation 4.


(4) $$\begin{array}{l} i_{z}=\sum_{x=1}^{d}IMF_{x}+e_{d}^{z} \end{array}$$

Compute a new 1D signal from the Gaussian white noise sequence shown in Equation 5. Where c-trials, *d*_*x*_(*z*)−Gaussian noise series, *i*_*x*_(*z*)−initial 1D signal.


(5) $$\begin{array}{l} i_{x}\left(z\right)=i_{z}+d_{x}\left(z\right)\;for\;x=1,2,3,..,c \end{array}$$

Evaluate the Eigen mode function of the frequency band as described in Equation 6. Let's assume EEM_D_−ensemble empirical mode decomposition, x, y-identified by frequency band. M-complete ensemble.


(6) $$Eℰ{ℳ}_{D}\left(y\right)=\frac{1}{c}\sum_{x=1}^{c}m_{xy}\left(z\right)$$

The adaptive filter used for filtering is calculated at the beginning of the procedure described in Equation 7. Where *i*[*d*]−uncorrelated with a reference signal, D-Noise, P*c*_*g*_ −Phonocardiogram.


(7) $$\begin{array}{l} i\left[d\right]=yPc_{g}+D \end{array}$$

Adaptive filters are standard and have a straightforward cost function. They generate a quadratic cost function with a global minimum for noise filtering. Calculate the noise in the reference signals between the auto-correlation matrix and cross-correlation vectors, as shown in Equation 8. Let's assume *the p*^*z*^−filter coefficient, N-noise filter, w=cross correlation matrix, M_*in*_−global minimum, *y*−cost function, and e-auto correlation matrix.


(8) $$Y=\{\begin{array}{c} R\left\{r^{2}\left[d\right]\right\}={\left(n\left[d\right]-j\left[d\right]\right)}^{2}\to M_{in}\\ R\left\{r^{2}\left[d\right]\right\}=ℛ\left\{n^{2}\left[d\right]\right\}-2P^{z}W+p+P^{3}e_{p} \end{array}$$

As demonstrated in Equation 9, the filter coefficients were computed for every iteration. Where μ_*r*_(*d*)−maximal amplitude


(9) $$\begin{array}{l} P\left(d+1\right)=p\left(d\right)+\mu_{r}\left(d\right)\;*\;i_{\left(d\right)} \end{array}$$

The upper bound is calculated as shown in Equation 10. As presented in Equation 11, the reference signal's mean power and step size can be estimated. Where C-measure the miss-adjustment, μ− size, *p*_*ow*_ −power.


(10) $$\begin{array}{l} \mu_{D}=\frac{2}{3\;*\;D.M_{ax}{\left(i\right)}^{2}} \end{array}$$

(11) $$\begin{array}{l} D=\frac{\mu }{2}\;*\;Dp_{ow}\left(i\right) \end{array}$$

Compute the convergence time as shown in Equations 12, 13 Where α− normalized step-size, ς−time, H(E)− eigenvalue, *m*_*d*_−condition number, λ(M_*ax*_), λ(M_*in*_)−minimum, and maximum normalized value,


(12) $$\begin{array}{l} \varsigma \approx \frac{1}{4\alpha }\;*\;H\left(E\right) \end{array}$$

(13) $$\begin{array}{l} H\left(E\right)=\frac{\lambda \left(M_{ax}\right)}{\lambda \left(M_{in}\right)}\;*\;m_{d} \end{array}$$

To achieve maximum probability estimation, the largest and smallest eigenvalues of the residual signal can be normalized by estimating the original one-dimensional signal across its frequency band or Eigen mode function. Through integrated empirical methods, it becomes possible to assess the frequency band of the distortion accurately.

Table 5 compares the performance of various pre-processing methods with diverse PCG datasets such as Pascal, Circor, and Physico- cardnet. The proposed EMDAMF method achieved 80.10% for Pascal, 85.34% for Circor, and 90.35% for the Physico-cardnet dataset. In contrast, existing filters failed to achieve high performance compared to the proposed method.

**Table 5:** Comparison performance of pre-processing methods.

**Methods/datasets**	***Z*-score**	**Min-Max scaling**	**EMDAMF**
Pascal	68.33	71.08	80.10
Circor	72.01	77.47	85.34
Physico- cardnet	78.19	81.35	90.35

### 3.3 Support Scalar Cardiac Impact Rate (S2CIR)

An infinite number of hyperplanes in the dataset can be detected by measuring the support vector using heart disease data for efficient classification. Furthermore, an optimal hyperplane can be evaluated using a quadratic kernel function and predicted heart disease data utilizing the maximum shadow width. The optimal hyperplane is found by dividing the data into two types. Regardless of their proximity to each group of objects, the support vector scalar optimal classifier can identify similar optimal generalization hyperplanes. A hyperplane can optimally partition the coordinate input set if the maximum distance between neighboring elements and the support vector impact rate is accurately split. In the binary classification of cardiovascular diseases, the bias and its weight vector can be estimated by computing the hyperplane through the class labels of the S2CIR technique, a process that is crucial for the SVM model's operations. The SVM model's hyperplane classification maximizes the margin during impact rates. The impact rate is calculated as the sum of distances to the nearest positive or negative event. Besides, the SVM model can estimate and predict the scalar vector impact rate by classifying heart disease data.

Assuming binary classification, heart disease is classified using the linear separation rule of training samples in Equation 13. Let's assume w-training sample, i-design matrix, j-binary class scalar vector,


(13) $$\begin{array}{l} \text{w}=\left\{H\left(i_{1},j_{1}\right),\left(i_{2},j_{2}\right),..,\left({\;𝔦}_{d},{𝔧}_{d}\right)\right\} \end{array}$$

Calculate the coordinate vector of the hyperplane using the binary classes, as indicated in Equation 14. Where r-function vector, *t, i, and β*− coordination of the hyperplane.


(14) $$\begin{array}{l} \text{r}\left(\text{i}\right)=\text{w}\left\{\left(\text{t,i}\right)+\beta \right\} \end{array}$$

Calculate the weight vector and bias between each hyperplane as shown in Equation 15. Let's assume p-weight vector, v-bias, x-class label.


(15) $$\begin{array}{l} \text{S.i}+\text{v}=0 \end{array}$$

As demonstrated in Equations 16, 17 hyperplanes are employed to categorize the training and testing heart disease data and approximate the prior function's kernel function. Where D-number of the training sample, h-kernel function, *i*_*x*_−input training sample, *j*_*x*_−matching class label, α_*x*_ −coefficient.


(16) $$\begin{array}{lll} \text{N}\left(\text{i}\right) & = & \text{sin}\left(\text{S.i}+\text{v}\right) \end{array}$$

(17) $$\begin{array}{lll} \text{N}\left(\text{i}\right) & = & \text{sin}\sum_{\text{x}=1}^{\text{D}}\alpha_{\text{x}}{\text{j}}_{x}\text{h}\left(\left({\text{i}}_{\text{x}},i\right)+v\right) \end{array}$$

Calculate the feature space coefficients from the input vector numbers of training samples as shown in Equation 18.


(18) $$\begin{array}{l} \sum_{\text{y}=1}^{\text{D}}\alpha_{\text{x}}{\text{j}}_{\text{x}}=0 \end{array}$$

Estimating the contribution of heart disease data by introducing a set of slack variables is illustrated in Equations 19, 20. Let's assume I_R_−impact rate, l−loss, ξ−slack variable, m- parameter, x-instance, L-normalization, S-weight vector, v-bias, *h*−dataset instance, W-sum of distance, z-vector.


(19) $$\underset{\text{s,v},\xi }{\min}\frac{1}{2}{\left\|\text{s}\right\|}_{2}^{2}+\text{m}\sum_{\text{x}=1}^{\text{h}}\xi_{\text{x}}\leftarrow {\text{I}}_{\text{R}}\;or\;ℒ$$

(20) $$\text{w.z}\left({\text{I}}_{\text{R}}\right)\{\begin{array}{c} {\text{v}}_{\text{x}}\left(\text{s},i_{x}+v\right)\geq 1-\xi_{x}\\ \xi_{x}\geq 0\;X=1,\ldots ..,\text{h} \end{array}\;$$

Therefore, Tables 6, 7 optimal features can be achieved by selecting different feature subsets, estimating the impact rate of cardiovascular disease, and introducing a set of slack variables to assess heart disease data.

**Table 6:** Heart sound signal frequency limits.

**Sound signal modulation**		**Frequency margins in Hz**		**Error adjustment**

**Murmur agonizes variables**		**Actual min range**	**Actual max range**	
Aortic regurgitation	AR	65≥, ++	395 ≤ , ––	±10.5, 0.5
Tricuspid regurgitation	TR	90≥, ++	400 ≤ , ––	±16.5, 0.1
Pulmonary regurgitation;	PR	90≥, ++	150 ≤ , ––	±15.7, 0.9
Patent ductus arteriosus;	PDA	90≥, ++	140 ≤ , ––	±20.9, 0.4
Mitral valve prolapse;	MVP	45≥, ++	90 ≤ , ––	±7.5, 0.5
Mitral stenosis	MS	40≥, ++	95 ≤ , ––	±6.7, 0.7
Mitral regurgitation	MR	45≥, ++	160 ≤ , ––	±9.0, 0.5
Atrial septal defect	ASD	60≥, ++	200 ≤ , ––	±25.5, 0.5
Aortic stenosis	AS	100≥, ++	450 ≤ , ––	±35.4, 0.3
Pulmonary stenosis	PS	150≥, ++	380 ≤ , ––	±27.1, 0.7
Normal heart frequency	NHF	100≥, ++	180 ≤ , ––	±20.5, 0.2

**Table 7:** Classified PCG sound signal disease labels.

**Label name**	**PCG frequency difference**	**Disease type**	
AS	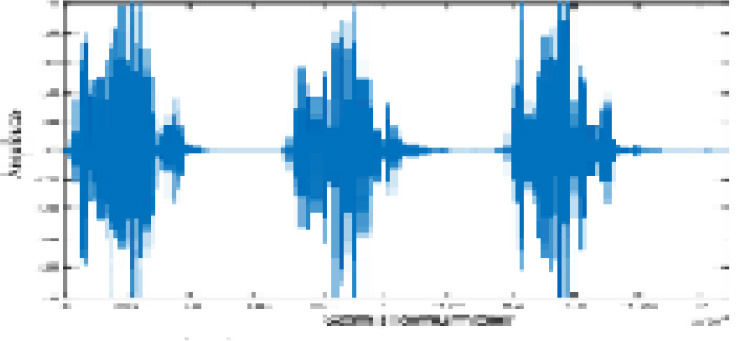	Aortic stenosis	
		100≥, ++	450 ≤ , ––
MR	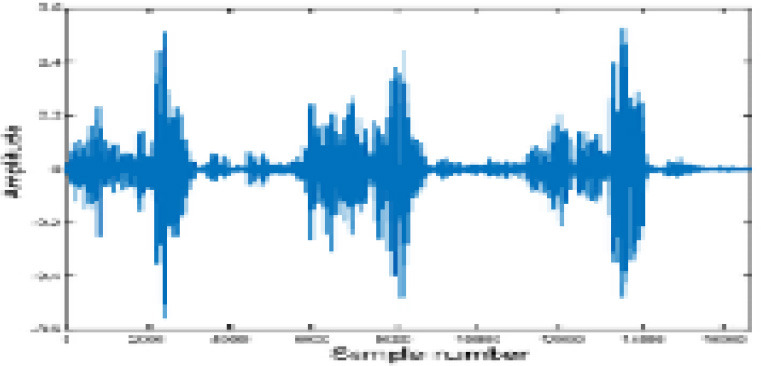	Mitral regurgitation	
		45≥, ++	160 ≤ , ––
MS	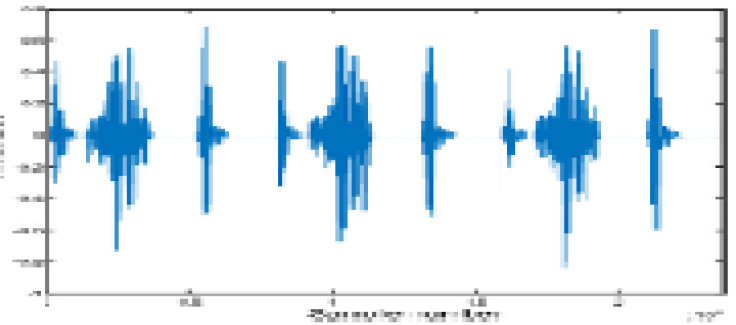	Mitral stenosis	
		40≥, ++	95 ≤ , ––
MVP	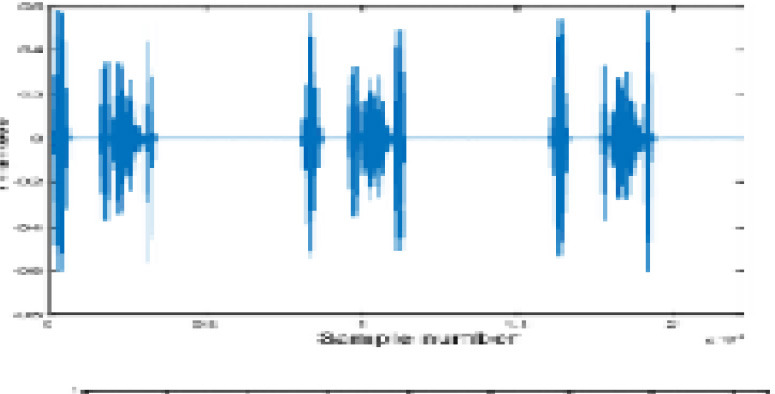	Mitral valve prolapse	
		45≥, ++	90 ≤ , ––
NHF	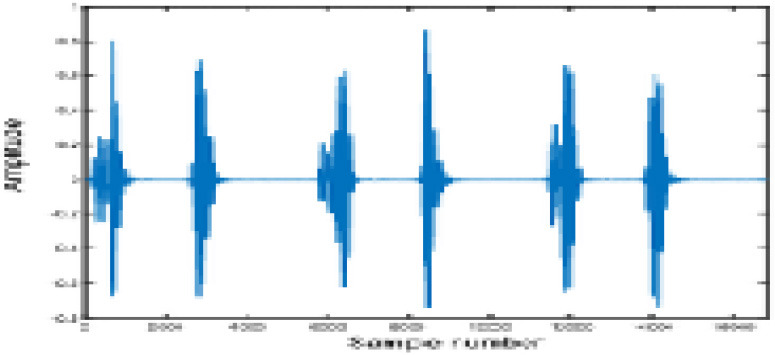	Normal heart frequency	
		100≥, ++	180 ≤ , ––

### 3.4 Linear vectorised particle swarm optimization

In this section, the linear vector particle swarm optimization method can be enhanced for classifying heart diseases and estimating their weights. This method provides a systematic approach to optimize the classification process, leading to more accurate and efficient results. The distance can also be selected by weighing the information provided by the attributes in linear vectors. After that, the attribute information is processed using linear vector distance calculation. The optimal particle processing time can be estimated by selecting the information attribute and multiplying the information gain using convergence behavior.

Similarly, linear vector performance can be improved by choosing values in categories related to heart disease. A search-based particle swarm optimization technique is employed to classify heart disease by comparing populations within the selected linear vector. The current optimal particle evaluation space is a possible solution within the linear vector PSO method. Furthermore, particle weights can be calculated to maintain cardiovascular data's homogeneity and heterogeneity effects. Moreover, greater inertial weights enhance global search efficiencies, whereas lesser ones enhance local ones. Similarly, the LV-PSO algorithm efficiently analyses the search space by adjusting the inertial weight with the acceleration factor. Consequently, the proposed LV-PSO method categorizes heart diseases based on the number of particles that regulate the parameters and ensure their balance in each iteration.

Equation 21 plays a crucial role in our method. It calculates the class labels by finding the number of class labels and choosing a weight vector corresponding to the unit value of each vector of the linear function. This step is significant as it forms the basis for the classification process, ensuring accurate and reliable results. Let's assume *the* K_*d*_−linear function, g-value, z-weight vector, and *a*_*g*_−training vector.


(21) $$\begin{array}{l} {K_{d}}_{g}=\left({\text{I}}_{\text{R}}\right)\leftarrow z_{1}\;*\;a_{g}+Z_{0} \end{array}$$

Equation 22 illustrates that the weight vector's difference between each data output unit's actual and predicted heart disease output values is computed. Furthermore, the error in each vector is estimated as described in Equation 23. Let's assumeDI_*d*_(*g*)−difference function, h-output unit, b-output value, q-error vector.


(22) $$DI_{g}\left(a\right)\;=(b_{g}-b_{a}^{'})2={\left(b_{a}-KE_{a}\right)}^{2}$$

(23) $$\begin{array}{lll} H & = & \frac{1}{q}\sum_{a=1}^{q}DI_{g}\left(a\right) \end{array}$$

Calculate the new weight unit by choosing the maximum number of iterations with the lowest learning rate starting from the alpha value shown in Equation 24. Let's assume R- -training vector, e-class, h-output unit, *z*_*h*_−weight of winning unit, and α−learning rate.


(24) $$H=\{\begin{array}{c} ifR=e_{h}then\;z_{h}=z_{h}\left(OL_{d}\right)+\alpha \left[a-z_{h}\left(O{ℒ}_{d}\right)\right]\\ ifℛ\neq e_{h}\;then\;3_{h}=Z_{h}\left(o\ell_{d}\right)+\alpha \left[a-3_{h}\left(O{ℒ}_{d}\right)\right] \end{array}$$

Compute the Euclidean distance of the information gain as shown in Equation 25. Let's assume *a*_*g*_− training vector class, C- Euclidean distance, G_*i*_−information gain, and g-class.


(25) $$\begin{array}{l} C\left(g,h\right)=\sqrt{{\left(a_{g}-z_{h}\right)}^{2}}\;*\;G_{i}\left(g\right) \end{array}$$

Equations 26, 27 below calculate particle size analysis by iterating particle velocities. Let's assume F−dimensional space, m-particle size, o−velocity, z-interval weight, ϕ_1_−local accelerations, ϕ_2_− Global accelerations, $m_{a}^{f}-$position of the particle, p-best position, K_*a*_(*p*) and *i*(*p*)−local and global best position,


(26) $$\begin{array}{lll} F & = & o_{a}^{f}\left(0\right)A\in \left\{m_{0},m_{1},{𝔪}_{2},\ldots ,M_{q}\right\},f\in \left\{0,1,..,f\right\} \end{array}$$

(27) $$\begin{array}{l} \sigma_{a}^{f}\left(p\right)=Zo_{a}^{f}\left(p-1\right)+\phi_{1}(K_{a}\left(p\right)-m_{a}^{f}\left(p-1\right)\phi_{2}i\left(p\right)\\ \;-m_{a}^{f}\left(p-1\right)) \end{array}$$

The control parameters are automatically chosen at each iteration depending on the swarm particle count. Equations 28, 29 below illustrate that random numbers between 0 and 2 can be computed to determine acceleration factors for local and global acceleration values. Where E-coefficient, E_1_ *and* E_2_−acceleration coefficient, $\frac{m_{q}}{8}-$particle generates a q/8 random number selection among acceleration coefficients, ϕ−accelerartion, q-number of swarm particles, T_1_ *and* T_2_−randiom number,


(28) $$E\;=\{\begin{array}{c} E_{1}={ℰ}_{1}^{1},E_{1}^{2},E_{1}^{3},..,E_{1}^{\frac{m_{q}}{8}}\;Where\;0\leq E_{1}\leq 2\\ E_{2}=E_{2}^{1},{ℰ}_{2}^{2},E_{2}^{3},..,E_{2}^{\frac{m_{q}}{8}}\;Where\;0\leq {ℰ}_{2}\leq 2 \end{array}\;$$

(29) $$\phi \;=\{\begin{array}{c} \phi_{1}=\sum_{q=1}^{\frac{m_{q}}{8}}{ℰ}_{1}.T_{1}\\ \phi_{2}=\sum_{q=1}^{\frac{m_{q}}{8}}E_{2}.T_{2} \end{array}$$

As shown in Equation 30, particles can be classified into three classes (i.e., low, medium, and high) based on estimating local and global acceleration values.


(30) $$\phi_{1},\phi_{2}=\{\begin{array}{c} ℒ=if\left(0\leq e_{1},e_{2}\leq 0.8\right)\\ ℳ=ℰ\ell seif\;\left(0.9\right)\leq e_{1},e_{2},\leq 1.2\\ ℋ=e\ell se\;\left(1.3\leq {ℰ}_{1},E_{2}\leq 2\right) \end{array}$$

For the inertia weight values, select three values between 0.4 and 0.9 and categorize them as low, medium, and high. Furthermore, low approximates 0.4, and high approximates 0.9 by averaging the other two values. At each iteration, the weight values are determined based on Equation 31. Let's assume o -value, $H_{o}-$high value, $M_{o}-$medium value, $L_{o}-$low value.


(31) $$I_{f}=\left\{ \begin{array}{l} \left(\phi_{1}\;\&\phi_{2}\in \ell \right)\;then\;w={ℋ}_{o}\\ \left(\phi_{1}\;\&\phi_{2}\in m\right)\;then\;w={ℳ}_{o}\\ \left(\phi_{1}\;\&\phi_{2}\in m\;\right)then\;w={ℒ}_{o} \end{array}\right.$$

As shown in Equation 32, the positions of the particles are computed by randomly choosing the number of acceleration factors for each particle position.


(32) $$\begin{array}{l} m_{a}^{f}\left(p\right)=m_{a}^{f}\left(p-1\right)+O_{𝔞}^{𝔣}\left(𝔭\right) \end{array}$$

Calculate a random number of particle-level fitness functions between the current fitness function and the selection coefficient estimate. Then, choose and update the optimal fitness function based on minimization or maximization, as in Equation 33. Let's assume $K_{𝔞y}-$local fitness value, $I_{P}-$global position, ${\mathrm{mi}}_{n}-$minimum value, ${m𝔞}_{𝔵}-$maximum value, $ɤ(m_{a}\left(p_{k}\right))-$current fitness function,


(33) $$if={ℐ}_{P}\{\begin{array}{c} i\left(u_{1}\right)\wedge Y\left(v_{1}\right)\;thenf_{1}=\alpha_{1}^{i}+\beta_{1}^{j}+\gamma^{1}\\ i\left(u_{2}\right)\wedge Y\left(v_{1}\right)\;thenf_{2}=\alpha_{2}^{i}+\beta_{2}^{j}+\gamma^{2} \end{array}$$

The random number at the particle level within the current fitness function can be approximated by analyzing the variance in each weight vector across the predicted output values for heart disease in each dataset.

**Figure 4:**
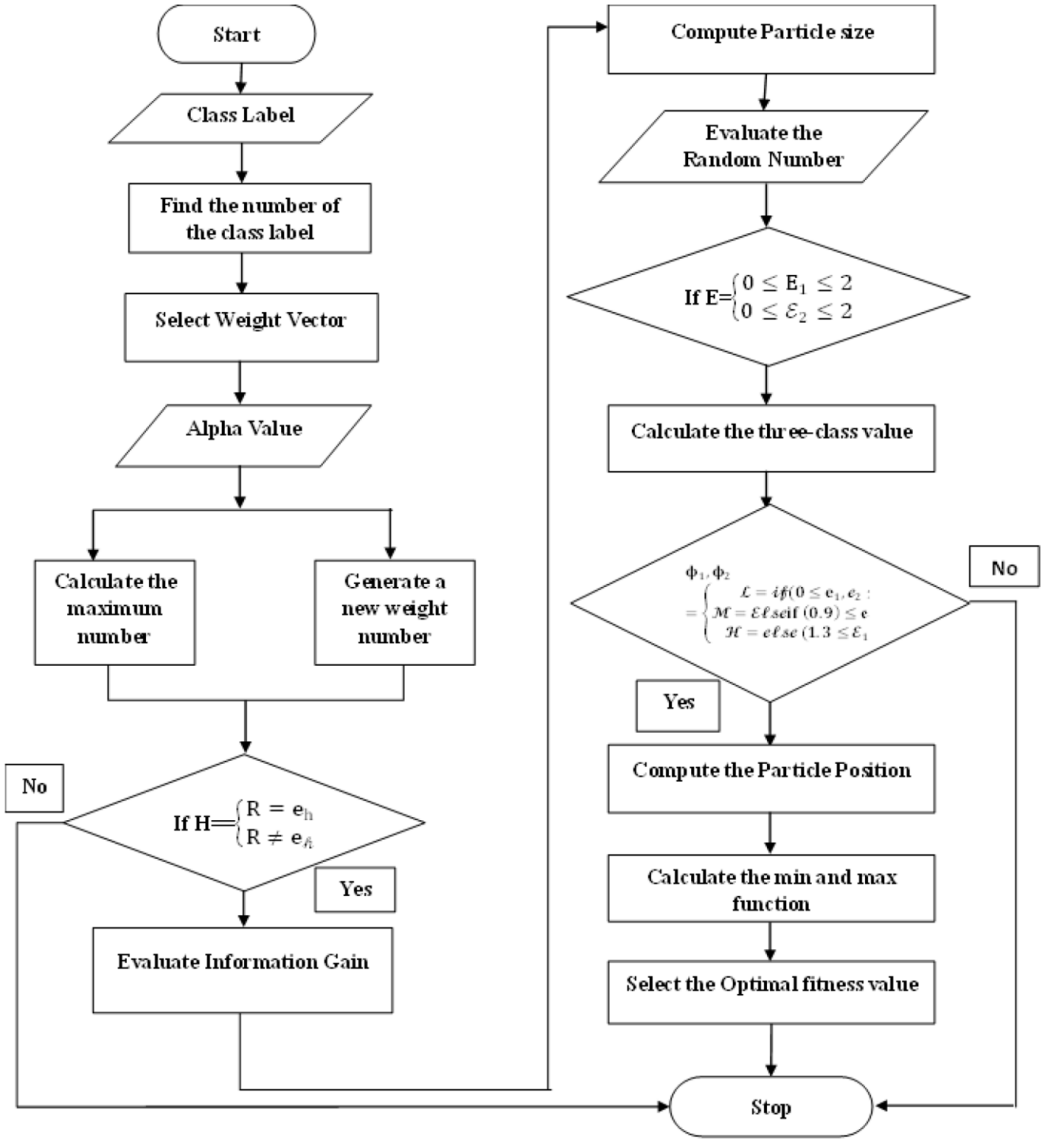
The LVPSO workflow.

A linear vector particle swarm optimization flow chart can classify particles into three categories based on selecting control parameters and estimating local and global acceleration values at each iteration. Furthermore, the best fitness function can be chosen based on minimum or maximum, and the best features can be selected and updated, as depicted in Figure 4.

Table 8 compares the performance of different feature selection methods and various PCG datasets. The proposed LV-PSO method yield is 81.24%, 86.085%, and 92.11% for Pascal, Circor and Physico-cardnet datasets, respectively. Similarly, the existing methods are Cuckoo Search Bio-inspired Algorithm (CSBA) and Particle Swarm Optimization (PSO) obtained less outcome performance than the proposed system.

**Table 8:** Comparison performance of pre-processing methods.

**Methods/datasets**	**CSBA**	**PSO**	**LV-PSO**
Pascal	70.08	74.13	81.24
Circor	73.61	77.24	86.08
Physico-cardnet	79.35	81.16	92.11

### 3.5 Fuzzy inference system (FIS)

In this section, fuzzy inference system methods use predefined fuzzy rules based on input maps and their related outputs. Moreover, accuracy can be evaluated for heart disease using rules defining relationships between fuzzy input and output sets. The input is fuzzy because it requires fuzzy values, and the output of the inference system is defuzzied to understand the user's production. Furthermore, the prediction and analysis of heart disease can be accomplished by utilizing a fuzzy model. Subsequently, its classification in each dimension can be segmented into rectangular subspaces by employing a predefined number of membership functions through the axis-parallel partitioning technique of the FIS method.

Fuzzy rules are “if-then” rules used in fuzzy logic systems to infer outputs based on the input variables. During forward propagation, the parameters obtained during training can be estimated by finding the least squares error. Similarly, gradient descent is utilized to analyse the parameters before training in the backward propagation process. After that, parameters for heart disease analysis are determined in forward and backward iterations.

As shown in Equation 34, fuzzy computers can predict heart disease as a function of a linear parameter using two rules: input and output. Let's assume i and j-input, $u_{1},v_{1}\;and\;U_{2},V_{2}-$fuzzy set, *g*_1_ *and g*_2_-fuzzy set, $\alpha_{1}^{i}+\beta_{1}^{j}+\gamma^{1}-$linear parameters.


(34) $$if={ℐ}_{P}\{\begin{array}{l} i(u_{1})\wedge Y(v_{1})\;thenf_{1}=\alpha_{1}^{i}+\beta_{1}^{j}+\gamma^{1}\\ i(u_{2})\wedge Y(v_{1})\;thenf_{2}=\alpha_{2}^{i}+\beta_{2}^{j}+\gamma^{2} \end{array}$$

Calculates input values and establishes membership function as a fuzzy layer. Equations 35, 36 demonstrate that fuzzification can be achieved using a Gaussian membership function. Where i and j-input, x-node, *m*_*x*_, ϱ_*x*_−parameter set, O−output layer, *m*_*x*_, −center of the curve, $\varrho_{x}-$gaussian member function,


(35) $$\begin{array}{lll} O_{1}^{x} & = & \mu U^{i}\left(i\right)\;x=1,2 \end{array}$$

(36) $$\begin{array}{lll} O_{1}^{x} & = & \;\mu U^{X}\left(i\right)=e^{\frac{{\left(i_{x}-m_{x}\right)}^{2}}{2_{x}^{\varrho^{2}}}} \end{array}$$

When the degree of fuzzy set membership equals zero, the trigonometric membership function is calculated as indicated in Equation 37. Let us assume w, r, and e –membership function, s-lower bound function, r-upper bound function, and e-center of the place degree of membership.


(37) $$\begin{array}{l} \mu U^{x}\left(i\right)=\left[\begin{array}{c} 0\;if\;𝔦\leq w\\ \frac{i-w}{r-w}\;if\;w\leq i\leq r\\ \frac{q-i}{e-r}\;if\;R\leq i\leq e\\ 0\;if\;i\geq e \end{array}\right] \end{array}$$

Calculate the appropriate bell-shaped membership function for the non-linear system defined by Equation 38. Furthermore, its membership function can be Gaussian or Bell-shaped. Where i and j-input variables, *u*^*x*^, *v*^*x*^, *m*^*x*^−bell membership function, u-width, v-slope, m-center of the bell. The learning mechanism of the training process determined these parameters.


(38) $$\mu {\left(U\Lambda V\right)}^{x}\left(i\Lambda j\right)=\{\begin{array}{c} \frac{1}{1+{\left|\frac{i-m_{x}}{u^{x}}\right|}^{2v_{x}}}\\ \frac{1}{1+{\left|\frac{i-m_{x}}{u^{x}}\right|}^{2V_{X}}} \end{array}$$

The input signal is amplified, and the output is sent via a straightforward amplifier with a set production. The output can be assessed by building the firing output of the product layer rule given in Equation 39. Let's assume *the p*_*x*_− weight of the fuzzy rule.


(39) $$\begin{array}{l} O_{2}^{x}=p_{u}\mu U_{x}\left(i\right)\mu V_{x}\left(j\right)=O_{1}^{x}\;*\;O_{2}^{x}\;x=1,2 \end{array}$$

As illustrated in Equations 40–42, the firing strengths calculated in the previous layer are evaluated and normalized at the fixed nodes. It multiplies the normalized values of the last layer and represents the first-order polynomial as a fuzzy representation. Calculate the maximum sum output of all input signals. Let us assume L-layer.


(40) $$\begin{array}{lll} O_{3}^{x} & = & \bar{p_{l}}=\frac{p_{x}}{\underset{x}{\sum }p_{𝔵}}\;X=1,2 \end{array}$$

(41) $$o_{4}^{x}\;=\bar{P_{\ell }}g_{x}=\bar{p_{l}}\left(w^{x}\left(i\right),R^{x}\left(j\right)+q_{x}\right)\;x=1,2$$

(42) $$\begin{array}{lll} O_{5}^{x} & = & g=\sum_{x}\bar{P_{l}}g_{x}=\frac{\underset{x}{\sum }p_{x}g_{𝔵}}{\underset{X}{\sum }{𝔭}_{X}} \end{array}$$

Let's assume $\bar{P_{l}}-$normalized weight, $w^{x},r^{x},e^{𝔵}-$consequent first-order polynomial parameter, and f-final output model.

As shown in Equation 43, the output can be determined using the centroid defuzzification method.


(43) $$\begin{array}{l} g=\frac{p_{1}g_{1}+p_{2}{𝔤}_{2}}{p^{1}+g^{1}}=\bar{P}g_{1}+\bar{P}g_{2} \end{array}$$

Equation 44 demonstrates that the calculation of heart disease can be represented as a linear combination of parameters in the fuzzy inference system's final output. Where *w*_1_, *r*_1_, *e*_1_ *and w*_2_, *r*_2_, *e*_2_linear combination of the parameters.


(44) $$g=\{\begin{array}{c} \frac{p_{1}}{p_{1}+P_{2}}g_{1}+\frac{P_{2}}{p_{1}+{\mathbb{P}}_{2}}g_{2}\\ \bar{P_{1}}g_{1}+\bar{p^{2}}g^{2}\\ \bar{P_{1}}\left(w_{1}\left(i\right)+r_{1}\left(j\right)+e_{1}\right)+\bar{P_{2}}\left(W_{2}\left(i\right)+r_{2}\left(j\right)+e_{2}\right)\\ \left(\bar{P^{1}}i\right)w_{1}\left(\bar{p^{1}}j\right)r_{1}+\bar{P^{1}}e_{1}+\left(\bar{P^{2}}i\right)w_{2}\left(\bar{p^{2}}j\right)r_{2}+\bar{P^{2}}e_{1} \end{array}$$

The parameters in the forward step in the fuzzy inference system can be determined using least-squares estimation. In backpropagation, each node's square signal error output can be propagated backward from the output layer to the input layer. Heart disease prediction can also be achieved through a fuzzy inference system by representing a linear combination of parameters.

**Figure 5:**
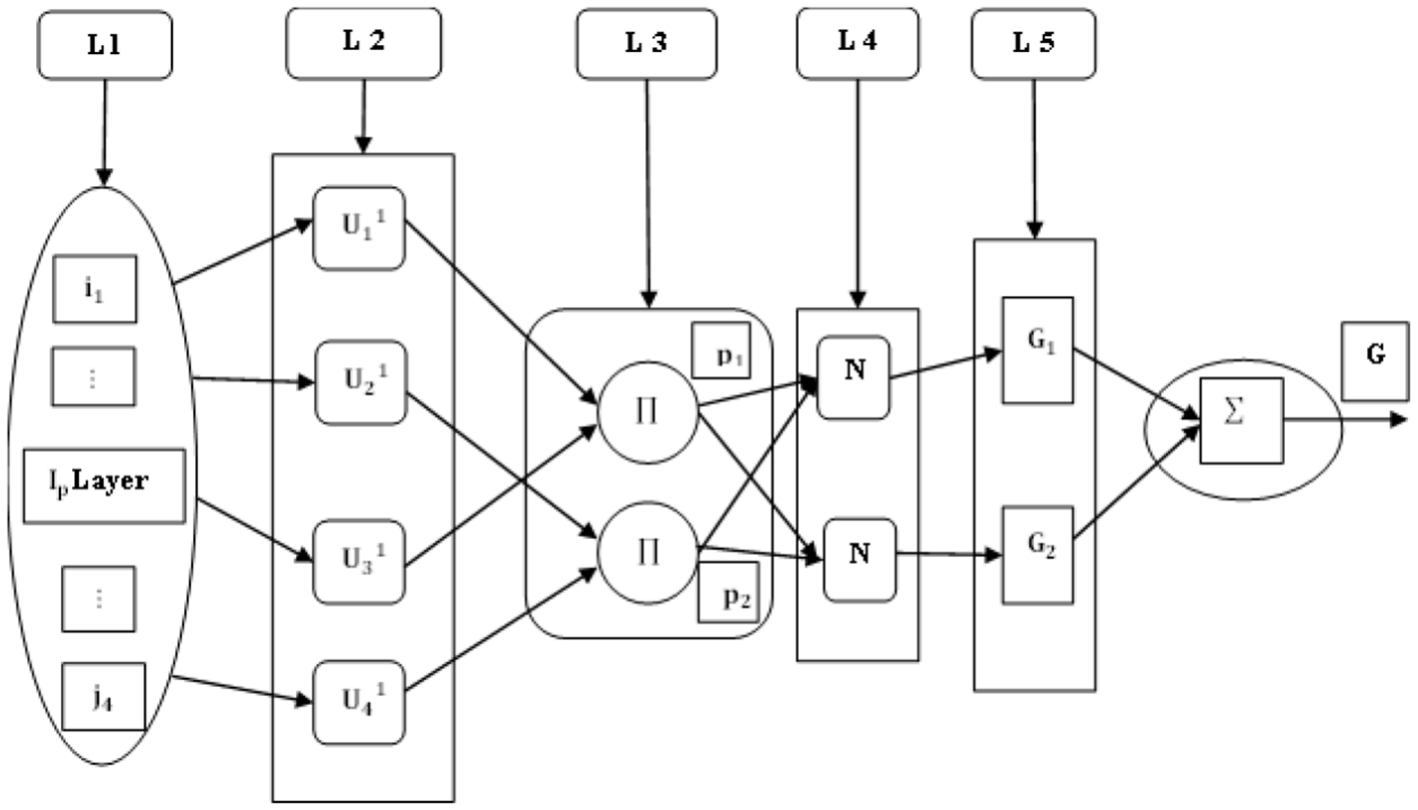
Fuzzy inference system.

The FIS uses a five-layer structure to forecast the maximum output, as presented in Figure 5. This includes L-layer, i and j as inputs, U_1_, U_2_, U_3_, *and* U_4_ as fuzzy subsets, Π as normalized nodes in layer 2, N as fixed nodes in layer 3, *p*_*i*_ as normalized weight and weight of the fuzzy rule, I_*p*_−input, and G_*i*_- fuzzy rule.

### 3.6. Xception Convolutional Neural Network

In this section, the proposed Xception Convolutional Neural Network method can be utilized to predict cardiovascular diseases. They use a feed-forward network on heart disease data for a CNN approach to data processing. CNN techniques incorporate feed-forward networks to enhance data processing systems' overall performance and dependability. Input data represented in dimensional scales can be combined with these filters to produce output feature maps. The XCNN approach preserves essential information at each process stage while reducing large feature maps to analyse heart disease.

The fuzzy logic structure in the proposed model is used to handle uncertainty in phonocardiogram (PCG) features by transforming the fuzzy inputs using membership functions. A set of fuzzy IF-THEN rules is then generated and trained by optimizing the membership function parameters integrated with XCNN (Explanatory Convolutional Neural Network), based on the number of convolutional layers, kernel sizes, and number of pooling layers. A typical XCNN architecture includes two to three transformable layers (e.g., 32 and 64 layers with kernel sizes of 5 and 3, respectively), followed by max-pooling, block normalization, ReLU implementation, a fully connected layer, and dropout before the final softmax or sigmoid output layer. Training is typically done using binary cross-entropy loss, Adam optimizer, learning rate 0.001, and about 50–100 epochs. The main missing component is a clear description of how the fuzzy system interfaces with the XCNN. This integration of the fuzzy inference system can act as a preprocessor, converting the extracted PCG features into high-level fuzzy risk scores, which are then combined with the CNN feature maps or provided as additional input to the fully connected layers of the XCNN.

Furthermore, it supports layers of important information or activities at each process stage. Input-output mapping is an essential feature of the activation functions in all neural networks.

Furthermore, the weighted sum of the neuron's inputs and dependencies can be used to evaluate the input values. The fully connected layer receives input from the final convolutional and pooling layer. Moreover, using the output layer to represent the preceding input layer, XCNN can identify and predict data related to heart diseases. The loss function in the output layer of the XCNN model considers the prediction error brought on by the training samples. After the fully connected layer, all vectors can be predicted utilizing binary classification in the SoftMax function to analyse heart disease data.

**Figure 6:**
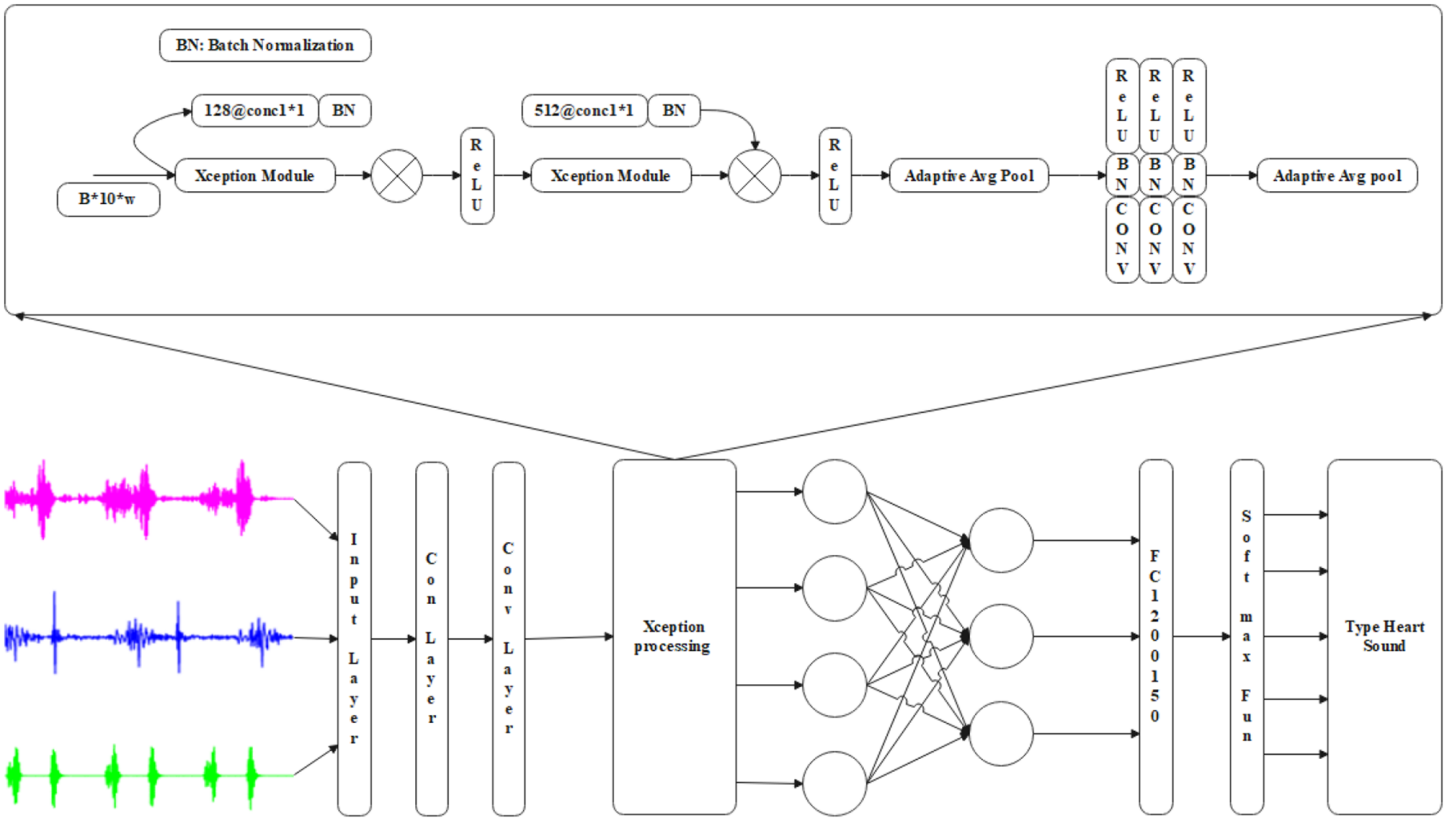
Xception processing based on heart sound prediction.

The results in Figure 6 demonstrate the effectiveness of using the Xception processing for heart sound prediction. This exception module approach accurately predicts cardiac data using input and convolutional layers combined with adaptive mean pooling layers. The dot product between the input weights is computed using the non-linear activation function of the convolutional layer output, as described in Equation 45. Where K^*h*^−feature map, f-feature, *p*^*h*^−weight, V-bias.


(45) $$\begin{array}{l} {\text{k}}^{\text{h}}\left(\text{g}\right)=g\left({\text{P}}^{\text{h}}\;*\;\text{i}+{\text{v}}^{\text{h}}\right) \end{array}$$

The sigmoid function in Equations 46, 47 evaluates input values of the processing function between 0 and 1. It also computes the output for the input of error backpropagation, an actual number between −1 and 1, as demonstrated in equation. R-back propagation, s-sigmoid function.


(46) $$\begin{array}{l} \text{g}{\left(\text{i}\right)}_{\text{s}}=\frac{1}{1+{\text{R}}^{-\text{i}}} \end{array}$$

(47) $$\begin{array}{l} 𝔤{\left(\text{i}\right)}_{\tanh}=\frac{{\text{r}}^{\text{i}}-r^{-i}}{{𝔯}^{𝔦}+{\text{r}}^{-\text{i}}} \end{array}$$

Converting all input values to positive numbers is a standard feature in the CNN context. As shown in Equation 48, the main advantage of ReLU is its lower computational burden. When the gradient is passed to the ReLU function, it can improve the regulation of neuron activation. Where Relu−rectified linear unit.


(48) $$\text{g}{\left(\text{i}\right)}_{ℛe\ell u}=\text{Max}\left(0,\text{x}\right)$$

Instead of disconnecting the ReLU negative input, this activation function is used to resolve the Dying ReLU problem. Utilize the ReLU to calculate the leakage coefficient value described in Equation 49. Let's assume the m-leakage factor, L-leakage.


(49) $$\text{F}{\left(\text{x}\right)}_{{\text{L}}_{\text{Relu}}}=\{\begin{array}{c} \text{i},\text{ if }>0\\ C_{i}\text{ i}\leq 0 \end{array}$$

Equations 50, 51 show that the noisy ReLU function converts ReLU into a noisy function by incorporating Gaussian distribution. Moreover, the leakage factor model in this procedure can be refined through training and assessed using parametric linear units. Let us assume D-noisy, c-max value, parametric linear, u-learnable weighted, j-Gaussian output distributed.


(50) $$g{\left(i\right)}_{{\text{N}}_{\text{Relu}}}\;=\text{m}\left(x+y\right),\;y\sim n\left(0,\sigma \left(\text{X}\right)\right)$$

(51) $$g\left(i\right){\text{w}}_{l}\;=\left\{\begin{array}{c} \text{i},\text{ if }g>0\\ {\text{u}}_{i}\;I\leq 0 \end{array}\right\}$$

The cross-entropy or Softmax loss function is commonly utilized in multiclass classification as an alternative to the Softmax and core error loss functions. The output is derived from the probability distribution function using the Softmax implementation in the output layer. The output class probability can be calculated as shown in Equation 52. Let's assume *p*_*i*_− preceding layer, N- number of neurons$s,\;and\;r^{u_{x}}-$non-normalized output.


(52) $$\begin{array}{l} {\text{W}}_{\text{x}}=\frac{\;{\text{r}}^{{\text{u}}_{\text{x}}}}{\sum_{\text{h}=\;1}^{\text{D}}{\text{r}}_{\text{u}}^{\text{h}}} \end{array}$$

Using Equation 53, compute the cross-entropy loss function. Where K-Hinge Loss Function.


(53) $$\begin{array}{l} \text{k}\left(\text{w,j}\right)=-\sum_{\text{x}}{\text{j}}_{x}\log\left(w_{x}\right)\;\text{x}\in \left[1,\text{D}\right] \end{array}$$

Below, Equation 54 presents the mathematical expression used to estimate the mean error Euclidean loss, commonly used in regression problems.


(54) $$\text{K}\left(\text{w,j}\right)=\frac{1}{2\text{D}}\sum_{\text{x}=1}^{D}{\left({\text{w}}_{\text{x}}-{\text{J}}_{x}\right)}^{2}$$

Equation 55 calculates the hinge loss function for binary and maximal edge-based classification to maximize the edge around the binary target class. Where J-desired output, c-margin dual objective class, *w*_*x*_−denote predicted output.


(55) $$\begin{array}{l} \text{K}\left(\text{w,j}\right)=\sum_{x=1}^{D}m_{\mathrm{ax}}\left(0,\text{c}-\left(2{\text{j}}_{\text{x}}-1\right){\text{r}}_{x}\right) \end{array}$$

Multi-classification with the ReLu function can predict vectors and analyse cardiac data. Utilizing ReLu in the output layer can also yield the probability distribution functions.

## 4 Evaluation and performance metrics

The results are tested under various categories in different datasets and feature limits accordingly; the PACAL database contains 656 heart sound recordings from de-identified patients by implementing the Pascal Challenge database. This dataset gathered from https://istethoscope.peterjbentley.com/heartchallenge/index.html. Furthermore, PCG signals were recorded at a sample rate of 4,000 Hz and ranged in duration from 1 to 30 s. After that, cardiac sounds can be captured in clinical and non-clinical contexts and categorized as heart sounds, standard sounds, murmurs, and artifacts. Similarly, static tones, noise, and premature seizures are characterized by implementing the Pascal challenge database. In addition, clinicians manually entered baseline heart sound levels into the Pascal Challenge database.

The Similar Physic Net dataset comprises 3,126 simultaneous recordings of PCG and ECG between 10 and 60 s. The dataset utilized in the Physio Net challenge included 2,435 cardiac recordings from 1,297 patients and was divided into training and testing sets. The PCG signal duration ranges from 8 to 312.5 s. Due to varying devices and sampling rates during data collection, each PCG signal was down-sampled to 2,000 Hz. Sounds from four sites (pulmonary artery, tricuspid valve, and mitral valve) were analyzed in healthy individuals and those with valvular heart conditions. An unbalanced data set is produced when the number of records in the training and test sets is much more than the number of anomalous records. In addition, the HSCT-11 database is the largest echocardiographic database in the field. In addition, it has heart sounds obtained from 206 individuals, which means that 157 individuals can be classified as male and 49 as female. After collecting data from each person, it can be predicted that the average queue length is 45 s, with a minimum of 20 s and a maximum of 70 s.

The Circor Digi Scope database, the largest publicly available heart sound dataset (https://www.kaggle.com/datasets/ bjoernjostein/the-circor-digiscope-phonocardiogram-dataset-v2), is a comprehensive resource for detailed analysis. It contains 5,282 recordings from various auscultation sites on the body, with most heart sounds divided into 200,464 recordings. The quality of these recordings was assessed by cardiac physiologists, leading to a thorough murmur characterization and classification. This detailed analysis examines the timing, grading, shape, quality, and location of auscultation, providing a wealth of information for researchers and clinicians.

The Heart Sound Shenzhen dataset, containing 845 PCG signal recordings from 170 individuals, is a comprehensive resource covering a wide range of heart diseases, including coronary artery disease, valvular disease, and congenital heart disease. The diversity of the HSS dataset significantly contributes to understanding the acoustic properties associated with various cardiac diseases. The PCG recordings in the HSS dataset were sampled at 4 kHz to ensure accuracy in the heart sound modeling.

A filter enables the PCG signal's decibel ratio based on heart sound variation for feature estimation as shown in Table 9. The empirical methods of signal analysis, using different dB levels to indicate signal types in PCG heart sounds, estimate the optimal accuracy of EMDAMF for disease type approximation. Various methods were employed to assess the accuracy prediction in analyzing AS heart sound variability within the PCG signal. The thorough evaluation included the SNR of de-noising heart signal wavelet rate −7.21, CEMD + median −9.43, and CEEMD −10.22. The AS heart disease category achieves an accuracy signal-to-noise ratio estimate of 11.54 in the EMDAMF approach. Similarly, heart diseases like MR, MS, MVP, and NHF did not yield precise ratios using the methods above. However, the EMDAMF model indicated higher accuracy ratios of 13.16, 15.22, 14.32, and 16.25 for heart conditions such as MR, MS, MVP, and NHF, respectively. This suggests the potential efficacy of the EMDAMF model in diagnosing specific heart sounds.

**Table 9:** SNR heart signal de-noising feature limits.

**Heart sound variation 0 dB**	**De-noising heart signals in dB SNR rate**.			

	**Wavelet**	**CEMD** + **median**	**CEEMD**	**Optimized EMDAMF**
AS	7.21	9.43	10.22	11.54
	Noise ratio ±0 *to* 4.5 difference ±1.5 variation			
MR	8.11	10.21	11.8	13.16
	Noise ratio ±0 *to* 5.5 difference ± 2.0 variation			
MS	9.87	11.43	13.86	15.22
	Noise ratio ±0 *to* 5.5 difference ±3.0 variation			
MVP	8.46	12.41	13.26	14.32
	Noise ratio ±0 *to* 6.0 difference ±2.7 variation			
NHF	10.98	13.28	14.76	16.25
	Noise ratio ±0 *to* 6.5 difference ±2.5 variation			

By dividing the dataset into “k” subsets (or folds), the model is trained on k-1 folds and validated on the remaining folds. This process is repeated “k” times, each fold serving as a validation set, and the results averaged to provide a more robust estimate of the model's accuracy.

**Figure 7:**
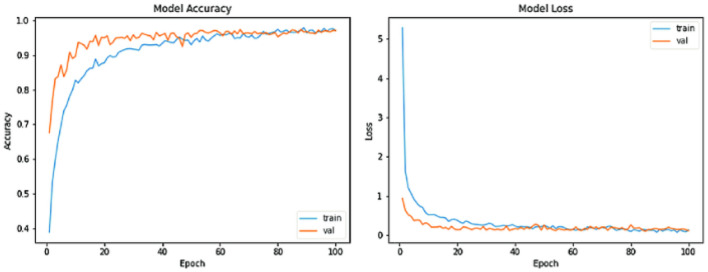
Testing accuracy and loss rate.

As shown in Figure 7, K5-fold cross-validation is a resampling technique that assesses classification algorithms on limited data samples. The results obtained from K5-fold cross-validation are considered less biased or unreliable compared to other methods like train/test separation. Moreover, K represents the number of folds divided into approximately equal-sized subsamples. Utilizing the proposed XCNN method within K5-fold Cross-Validation showcases a notable enhancement, with a remarkable 92.36% increase in folds employed for a random distribution aimed at accuracy prediction. This substantial increase is a testament to the XCNN method's impressive performance. Despite the significant improvement, the error rate-7 associated with the XCNN method is low.

**Figure 8:**
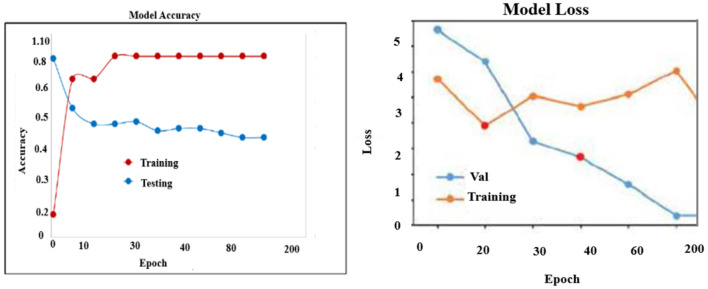
Ten fold cross validation.

As shown in Figure 8, compared to other methods such as train-test splitting, the results of 10-fold cross-validation indicate that the number of folds is divided into approximately equal-sized sub-samples. Additionally, the accuracy of 10-fold cross-validation is significantly improved to 93.6% in randomly distributed folds used for prediction, thereby substantially enhancing the performance of the proposed XCNN method in fold cross-validation. A 10-fold cross-validation was used to determine the optimal value, thereby improving the reliability of the model results.

As illustrated in Table 10, using datasets like PASCAL, Circor Digi scope, Physio Net Challenge, HSS, and HSCT dataset, sourced from various datasets, can yield precise estimations. Additionally, the total count of recordings of these sounds within each dataset is delineated, for instance, PASCAL-656, Circor Digi scope-5282, Physio Net Challenge-3126, HSS-845, MVP-206, and so forth. Furthermore, cardiac recordings can be used to identify features such as NHF, AS, MR, MS, MVP, and errors, which can be used to predict the overall accuracy of the dataset.

**Table 10:** Multi label dataset description.

**Dataset**	**Total**	**NHF**	**AS**	**MR**	**MS**	**MVP**	**Error**
PASCAL	656	256	129	89	92	81	9
Circor Digi scope	5,282	2,347	1,788	850	196	91	10
PhysioNet challenge	3,126	1,753	879	273	128	87	6
Heart Sounds Shenzhen (HSS)	845	404	213	91	76	53	8
Hematopoietic stem cell transplantation (HSCT)	206	56	49	39	37	18	7

The training and testing scheme analysis, as depicted in Table 11, implicates using the proposed method to analyse data from sources such as Heart Sound PASCAL, Circor Digi Range, Physio Net Challenge, HSS, and HSCT. Together, the accuracy of training and test outcomes is anticipated through the application of various technologies, including DNN, XGBoost, RNN, SMOTE, EMD, LMS, KNN, A-BLSTM, and MLP, all of which are derived from the preceding method—subsequently, the proposed method FIXCNN is employed for processing the provided heart sound dataset and forecasting accuracy. Furthermore, prior techniques analyzed DNN-81.6%, XGBoost-83.4%, RNN-84.6%, SMOTE-82.63%, EMD-88.18%, LMS-85.4%, KNN-89.2%, A-BLSTM-86.09%, and MLP-evaluates the accuracy of such heart sounds as 87.56%, and the suggested XCNN approach has the highest accuracy potential of 95.23%. This approach provides the ability to fully evaluate the predictive accuracy of the selected technique in the context of heart sound analysis, instilling confidence in the thoroughness of the evaluation process.

**Table 11:** Comparison of methods under testing and validation accuracy.

**Method**	**PASCAL**			**Circor Digi scope**			**PhysioNet challenge**			**HSS**			**HSCT**		

	**Train**	**Test**	**Acc**	**Train**	**Test**	**Acc**	**Train**	**Test**	**Acc**	**Train**	**Test**	**Acc**	**Train**	**Test**	**ACC**
DNN	402	254	81.6%	3,825	1,457	73.17%	2,459	667	75.34%	654	191	77.46%	132	74	78.25%
XGBoost	517	139	83.4%	4,434	848	73.46%	1,837	1,289	74.29%	661	184	76.19%	147	59	77.87%
RNN	533	123	84.6%	4,048	1,234	74.01%	2,131	995	75.67%	564	281	77.42%	128	78	78.46
SMOTE	487	129	82.63%	3,650	1,632	77.64%	2,143	908	79.2%	678	167	78.36%	144	62	89.2%
EMD	547	109	88.18%	3,825	1,457	78.31%	2,547	579	89.25%	731	114	77.56%	108	98	81.23%
LMS	519	137	85.4%	4,784	498	81.12%	2,173	953	83.12%	728	117	84.56%	132	74	85.6%
KNN	482	174	89.2%	3,388	1,894	79.3%	2,154	1,062	85.6%	671	174	79.26%	157	49	88.25%
A-BLSTM	514	142	86.09%	4,358	924	85.26%	1,895	1,231	82.4%	719	126	86.3%	139	67	79.38%
MLP	432	224	87.56%	4,501	781	89.23%	2,653	473	88.26%	964	81	86.36%	129	77	81.63%
XCNN	491	165	95.08%	3,514	1,768	91.3%	1,986	1,140	92.56%	709	136	93.69%	141	65	90.8%

**Figure 9:**
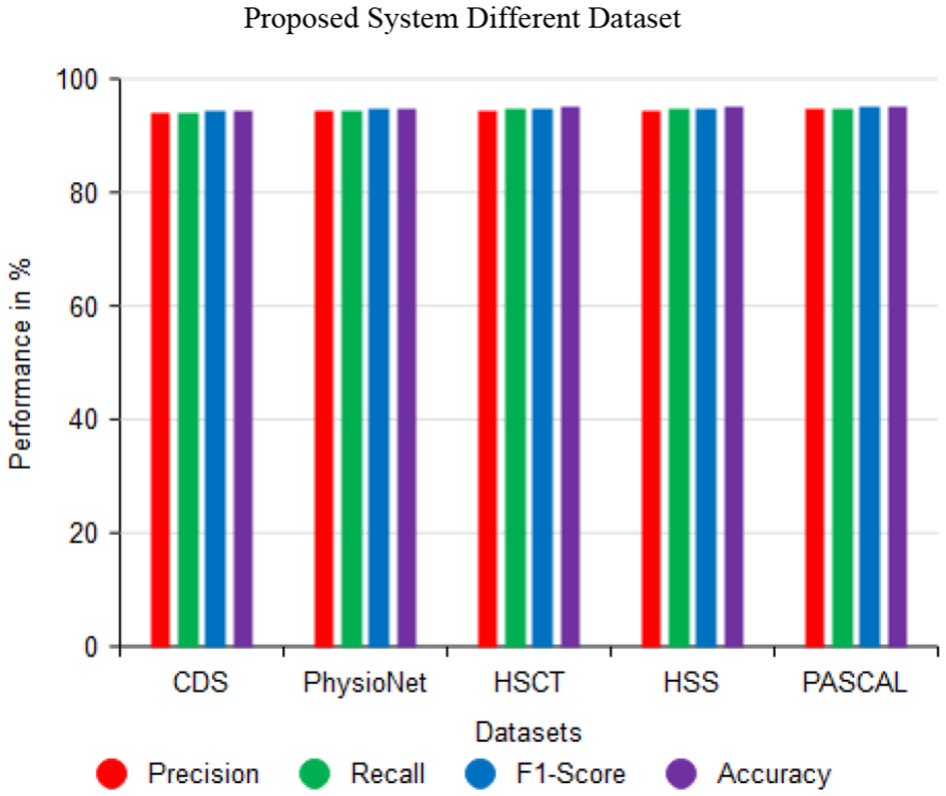
Impact of proposed system in different dataset.

As indicated in Figure 9, the proposed XCNN method can accurately predict the cardiac sound PCG signals using datasets such as Circor Digi scope, Physico-Net Challenge, HSCT, HSS, and PASCAL. Moreover, the PCG signal performance-based assessment of precision, recall, F1 score, and precision can accurately predict heart sound predictions. Then, Circor DigiScope-94.49%, Physico-Net Challenge-94.85%, HSCT-94.91%, HSS-95.01, and PASCAL-95.08% improved the accuracy of each performance measure on the heart sound data. Thus, by assessing the PCG signal's efficacy on cardiac sounds, the accuracy of these has been improved.

**Figure 10:**
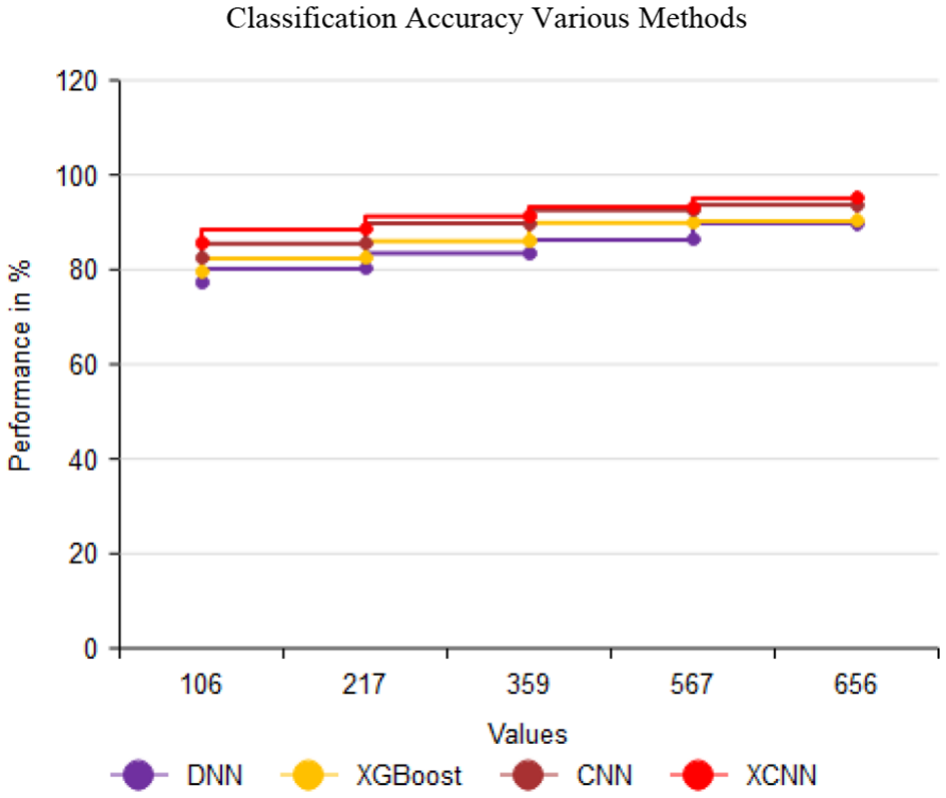
Comparison of various methods in classification accuracy.

The XCNN met hod demonstrates the capability to accurately predict the performance of cardiac sound PCG signals using datasets illustrated in Figure 10 and Table 12. This has significant practical implications, as it outperforms previous methodologies employing DNN, CN N, and XGBoost for heart sound accuracy detection. The manipulated versions of DNN, CNN, and XGBoost exhibited enhanced accuracies of 89.78%, 90.23%, and 93.69%, respectively. However, the XCNN method achieved the highest accuracy rate of 95.08%, establish ing itself as a promising method for precise heart sound analysis and offering new possibilities for healthcare technology.

**Table 12:** Comparison of performance accuracy attained.

**Methods**	**LSTM-RNN**				**CNN**				**LVPSO-FIXCNN**			
**Dataset**	**Precision**	**Recall**	**F1 score**	**Accuracy**	**Precision**	**Recall**	**F1 score**	**Accuracy**	**Precision**	**Recall**	**F1 score**	**Accuracy**
Pascal	78.3	86.2	79.5	80.1	82.3	82.1	84.3	86.1	91.6	92.5	94.3	95.2
circor	81.4	83.2	80.5	82.8	84.1	85.3	84.8	85.4	92.3	93.1	95.8	94.1
Physio-cardnet	79.5	81.2	82.4	83.1	85.8	83.9	86.1	87.1	91.4	93.1	96.1	95.6

**Figure 11:**
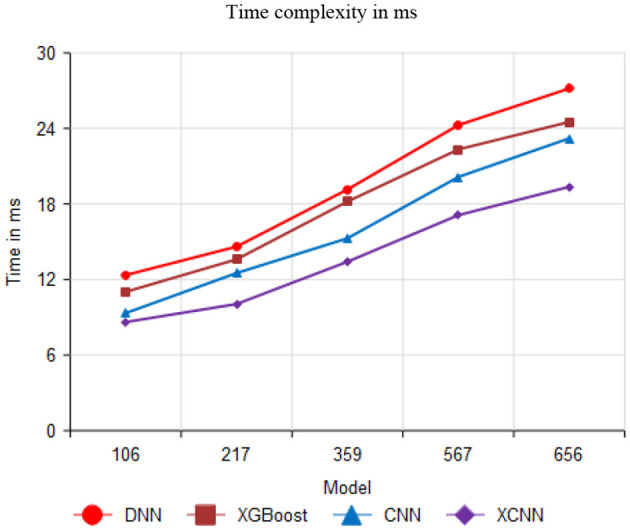
Comparison of time complexity.

Figure 11 compares time complexity performance with 106, 217, 359, 567, and 656 values. The proposed method attains 19.36 ms for 656 values; similarly, the existing method attained 27.17 ms, 24.51 ms, and 23.21 ms for DNN, XGBoost, and CNN, respectively.

**Figure 12:**
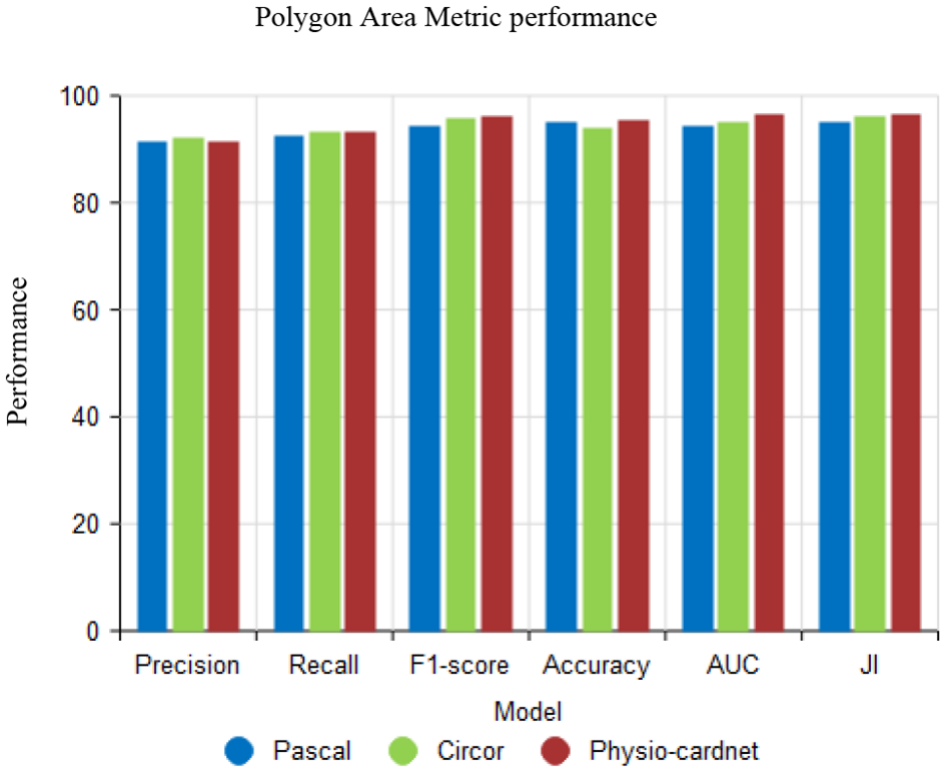
Performance of polygon area metric.

Figure 12 shows the Polygon Area Metric (PAM) performance using the proposed XCNN method. In the analysis, PAM is user metrics are precision, recall, F1-score, accuracy, AUC and Jaccard Index (JI). The proposed method attains better results using various datasets like Pascal, circor and physio-cardnet.

**Figure 13:**
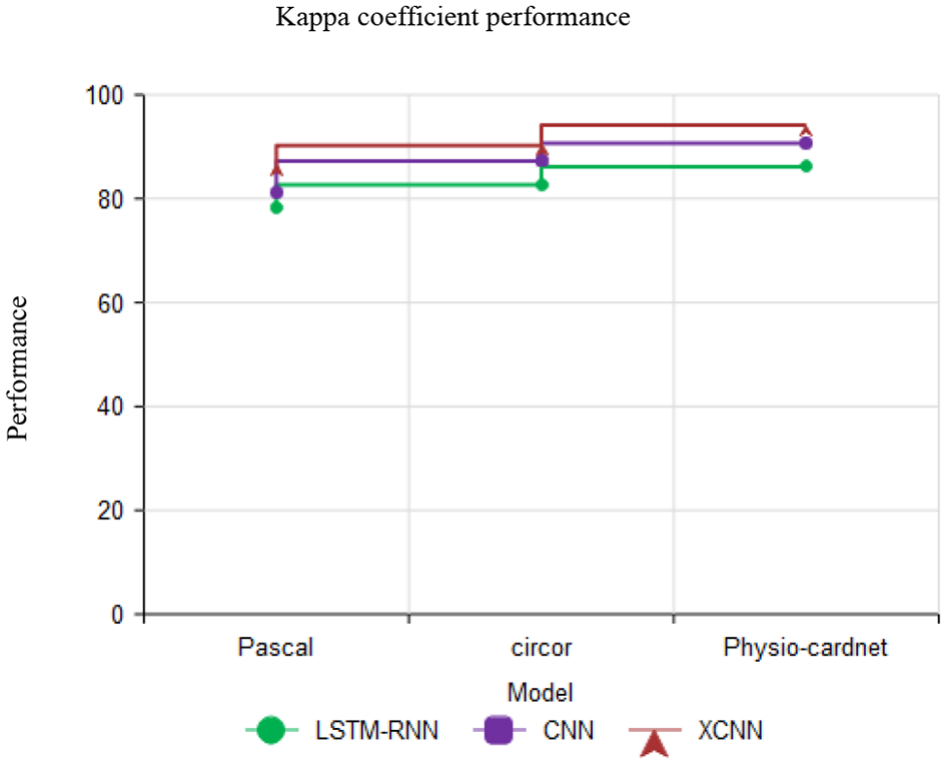
Comparison of kappa coefficient performance.

Figure 13 describes the Kappa coefficient performance with various datasets like pascal, circor and physio-cardnet. The kappa coefficient estimate the agreement among classification and truth values in the dataset. The proposed method obtains the high kappa coefficient performance than other methods.

### 4.1 Discussion

This study, which meticulously uses digital heart rate recordings and state-of-the-art ML algorithms, is designed to identify valvular heart disease (VHD) in the general population, including instances with no symptoms and intermediate phases of the disease. An RNN was trained to predict heart murmurs using annotated recordings from a digital stethoscope at four auscultation levels in 2,124 participants. The predicted sounds were then utilized to predict VHD detected by ECG.

Our research has provided specific and informative data on the accuracy of aortic stenosis (AS) detection. At the lower curve, we observed a 90.9% accuracy, 94.5% specificity, and an AUC value of 0.979 (CI: 0.963–0.995) for mild AS identification, with an accuracy of 0.993 (CI: 0.989–0.997). The AUC values for moderate or high aortic arch and mitral regurgitation (AR and MR) were 0.634 (CI: 0.565–703) and 0.549 (CI: 0.506–0.593), respectively, which increased to 0.677 and 0.766 with the inclusion of the variable.

Variability measures such as standard deviation (eg, precision = 91.2% ± 2.3%) and 95% confidence interval [AUC = 0.85 (0.83–0.91)] were frequently missing. These metrics are important for assessing the model's consistency and generalizability across different cross-validation combinations or test sets. The AUC for predicting symptomatic events was higher for AR and MR, 0.756 and 0.711, respectively. Incorporating screening for symptomatic regurgitation or stenosis, the AUC was 0.86, with 97.7% of AS cases (*n* = 44) and all 12 MS cases detected.

**Figure 14:**
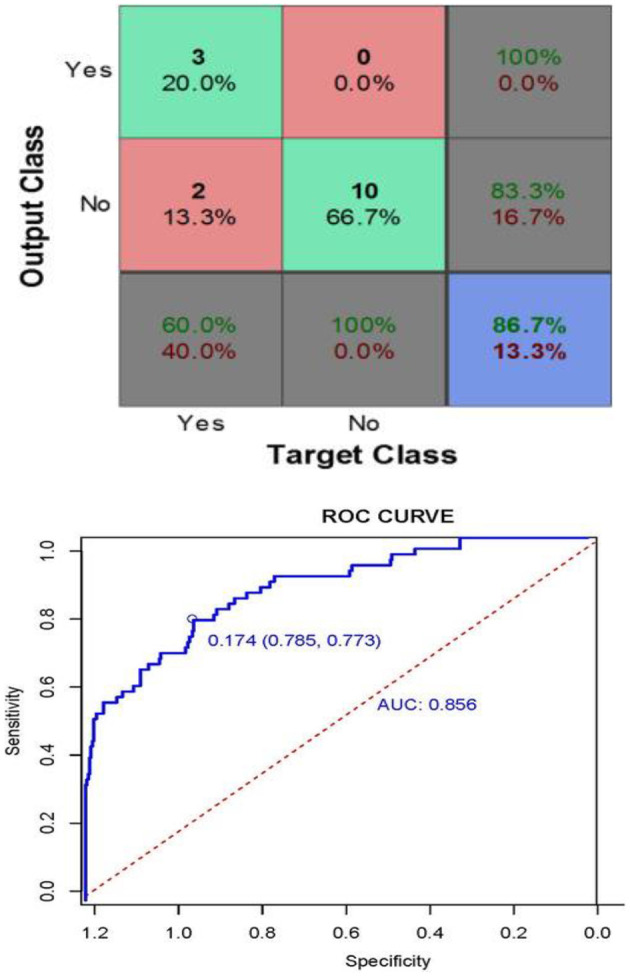
Confusion matrix, ROC, and AUC performance using confusion matrix.

Figure 14 defined as, This matrix describes the performance of the neural network model in terms of target class and output class values. The Curve Analysis (CA) for the model revealed that when the threshold probability of an individual was between 20% and 95%, application of this model to predict the heart disease prediction analyzing the AUC values of 0.856 [95% CI: 0.804, 0.908; *P* < 0.001]. The results showed that the test *p* = 0.098. According to the maximum principle of the index, the optimal cut-off value of AUC was 0.174, in which the accuracy was 77.3% and the sensitivity was 78.5%, indicating that the model had a good fit and a good fit with the data (Figure 13). After 2,000 recordings resampling iterations, the AUC was 0.852, showing that the model had good ability.

## 5 Conclusions

To conclude, the proposed system achieves high performance under various testing performances, proving that the proposed system achieves higher detection accuracy than the existing systems. This work uses the feature selection and classification approach to extract the heart sound signal contaminated by noise and murmur into a set of single-component signals to classify the result effectively. Furthermore, the most appropriate Intrinsic Mode Functions (IMF) can be selected to represent the undistorted fundamental heart sound signal. After that, EMDAMF can detect the presence of murmurs in the heart sound signals utilizing the PCG signal frequency limits for feature selection. Furthermore, the EMDAMF algorithm theoretically offers improved spectral separation compared to the EMD method while also managing the issue of mode combining present in the EMD method. In conclusion, creating an LV-PSO based on FIXCNN for early heart risk prediction signifies progress in healthcare technology. Furthermore, the accuracy performance evaluation of previous methods is presented: CNN−87.23%, XGBoost−90.17%, and RNN−86.18%. The proposed method improves the precision performance of the XCNN technique to 95.08%. This approach could revolutionize the diagnosis and treatment of heart disease by combining advanced ML techniques with innovative pre-processing methods, saving lives and improving patient outcomes.

## Data Availability

The original contributions presented in the study are included in the article/supplementary material, further inquiries can be directed to the corresponding authors.
